# Genome Organization of a New Double-Stranded RNA LA Helper Virus From Wine *Torulaspora delbrueckii* Killer Yeast as Compared With Its *Saccharomyces* Counterparts

**DOI:** 10.3389/fmicb.2020.593846

**Published:** 2020-11-23

**Authors:** Manuel Ramírez, Rocío Velázquez, Matilde Maqueda, Alberto Martínez

**Affiliations:** Departamento de Ciencias Biomédicas (Área de Microbiología), Facultad de Ciencias, Universidad de Extremadura, Badajoz, Spain

**Keywords:** yeast, *Torulaspora*, killer, virus, dsRNA LA genome, high-throughput sequencing, sequence comparison

## Abstract

Wine killer yeasts such as killer strains of *Torulaspora delbrueckii* and *Saccharomyces cerevisiae* contain helper large-size (4.6 kb) dsRNA viruses (V-LA) required for the stable maintenance and replication of killer medium-size dsRNA viruses (V-M) which bear the genes that encode for the killer toxin. The genome of the new V-LA dsRNA from the *T. delbrueckii* Kbarr1 killer yeast (TdV-LAbarr1) was characterized by high-throughput sequencing (HTS). The canonical genome of TdV-LAbarr1 shares a high sequence identity and similar genome organization with its *Saccharomyces* counterparts. It contains all the known conserved motifs predicted to be necessary for virus translation, packaging, and replication. Similarly, the Gag-Pol amino-acid sequence of this virus contains all the features required for cap-snatching and RNA polymerase activity, as well as the expected regional variables previously found in other LA viruses. Sequence comparison showed that two main clusters (99.2–100% and 96.3–98.8% identity) include most LA viruses from *Saccharomyces*, with TdV-LAbarr1 being the most distant from all these viruses (61.5–62.5% identity). Viral co-evolution and cross transmission between different yeast species are discussed based on this sequence comparison. Additional 5′ and 3′ sequences were found in the TdV-LAbarr1 genome as well as in some newly sequenced V-LA genomes from *S. cerevisiae*. A stretch involving the 5′ extra sequence of TdV-LAbarr1 is identical to a homologous stretch close to the 5′ end of the canonical sequence of the same virus (self-identity). Our modeling suggests that these stretches can form single-strand stem loops, whose unpaired nucleotides could anneal to create an intramolecular kissing complex. Similar stem loops are also found in the 3′ extra sequence of the same virus as well as in the extra sequences of some LA viruses from *S. cerevisiae*. A possible origin of these extra sequences as well as their function in obviating ssRNA degradation and allowing RNA transcription and replication are discussed.

## Introduction

Most killer yeasts secret a killer toxin encoded by the positive strand of medium-size (about 2 kb) dsRNA of M viruses. Different types of M viruses have been described, each one encoding a specific killer toxin (Schmitt and Tipper, 1995; Rodríguez-Cousiño et al., 2011; Ramírez et al., 2017; Vepštaitë-Monstavičė et al., 2018). Each killer yeast contains only one type of M virus together with a large-size (about 4.6 kb) helper dsRNA virus (V-LA) that is required to maintain stability of the former and for its replication. V-LA provides the capsids and polymerase required to separately encapsidate, transcribe, and replicate both the LA and M dsRNAs. The M dsRNA contains some stem loops that mimic LA dsRNA signals required for packaging and replication (reviewed by Schmitt and Breinig, 2006). Two proteins are encoded in the V-LA genome—the coat protein (Gag), and a fusion protein translated by a -1 ribosomal frameshifting mechanism (Gag-Pol) that contains the polymerase activities required for virus propagation (Icho and Wickner, 1989; Dinman and Wickner, 1992; Fujimura et al., 1992; Park et al., 1996). Viral RNA packaging and replication require some *cis* signals located in the 3′-terminal regions of the positive strands (Wickner et al., 1995; Rodríguez-Cousiño et al., 2011). It has been proposed that the signal for transcription initiation of the mRNA (positive strand) is located in the first 25 nucleotides of the 5′ end of the same strand, involving the terminal 5′-GAAAAA motif itself (Thiele and Leibowitz, 1982; Thiele et al., 1982; Fujimura et al., 1990; Rodríguez-Cousiño et al., 2011), which is 3′-CTTTTT in the negative-strand template.

Based on the sequence identity and type of the accompanying M virus, several V-LA isotypes found in yeast species included in the *Saccharomyces sensu stricto*: *S. cerevisiae*, *S. paradoxus*, *S. kudriavzevii*, and *S. uvarum* (Table 2). These viruses share 73–93% identity in viral genome nucleotide sequences and 87–99% identity in amino-acid Gag-Pol sequences. The identity among viruses from *S. paradoxus* strains seems to depend on their geographical location. Although sequences of *S. paradoxus* LA viruses are found to be more homogeneous than their *S. cerevisiae* counterparts, two separate clusters have been proposed for the former: one including SpV-LA66, SpV-LA28, and SpV-LA21, and the other containing the remaining LA viruses from *S. paradoxus*. The data available does not allow any cluster to be defined for *S. cerevisiae* LA viruses. Despite this, all the *Saccharomyces* LA viruses investigated thus far conserve the essential features found in the first *S. cerevisiae* LA virus to be described (ScV-LA1-original), such as the frameshift region and encapsidation signal (Rodríguez-Cousiño et al., 2017; Vepštaitë-Monstavičė et al., 2018).

Based on the association of some V-LA isotypes with specific M viruses in the same type of killer yeast, it has been suggested that each viral pair co-evolved with each other in their natural environment (Rodríguez-Cousiño et al., 2013). This suggestion is reinforced with the finding that neither ScV-LA nor ScV-LAlus show helper activity for M2, while the specific presence of ScV-LA2 is required for M2 maintenance in the same genetic background (Rodríguez-Cousiño and Esteban, 2017). However, ScV-LAlus helper activity for M2 has being found by other authors in another *S. cerevisiae* strain (Lukša et al., 2017). Sequence identity variations among these virus isotypes seem to depend on the geographical location of the host, it has also been suggested that V-LA cross-species transmission occurs between different yeast species living in the same habitat (Rodríguez-Cousiño et al., 2017).

The yeasts included in the *Saccharomyces sensu stricto* taxon (*S. cerevisiae*, *S. uvarum*, *S. paradoxus*, *S. mikatae*, *S. kudriavzevii*, *S. arboricola*, and *S. eubayanus*) have very similar genomes and a close phylogenetic relationship. Among them, *S. paradoxus* is considered to be the closest relative to *S. cerevisiae* (Kurtzman and Robnett, 2003; Scannell et al., 2011). Therefore, in the wild, hybridization can be expected among these yeast species. Indeed, natural hybrids have been found between *S. cerevisiae* and *S. eubayanus*, *S. uvarum* and *S. eubayanus*, and between *S. cerevisiae* and *S. kudriavzevii* (Hittinger, 2013). Most species of the *sensu stricto* taxon are frequently associated with human activities such as bread baking and alcoholic fermentations. These circumstances may favor horizontal cross-species transmission of killer viruses by yeast mating. Even some species that seem to live in different natural habitats, such as *S. cerevisiae* (mainly involved in food fermentation processes) and *S. paradoxus* [mainly present in the wild, associated with oak trees or the surrounding soil (Liti et al., 2009)], can mate in close-to-wild laboratory conditions and transfer killer viruses from one to another (Rodríguez-Cousiño et al., 2017). As these viruses are cytoplasmically inherited and spread horizontally by cell-cell mating or heterokaryon formation (Wickner, 1991), the presence of a specific virus isotype in different yeast species indicates that these yeasts may be able to mate in the wild.

The phylogenetic relationship between *Torulaspora* and *Saccharomyces* is not as close as that among the yeasts of the *sensu stricto* group. However, *T. delbrueckii* is quite similar to *S. cerevisiae* in the sense that both are among the best fermentative yeasts for biotechnological applications, and can share the same habitat in several ecosystems such as the spontaneous fermentations of bread dough, beer, wine, and different fruits (Kurtzman and Robnett, 2003; Kurtzman, 2011a,b). *T. delbrueckii* is probably the most used non-*Saccharomyces* yeast in winemaking. Killer Kbarr1 *T. delbrueckii* kills all known *S. cerevisiae* killer strains and other non-*Saccharomyces* yeasts. The Kbarr1 phenotype is encoded with the M virus (TdV-Mbarr1) that depends on an LA virus (TdV-LAbarr1) for its maintenance and replication. The TdV-Mbarr1 dsRNA sequence organization is quite similar to that of the *S. cerevisiae* killer M dsRNAs: a 5′-end coding region followed by an internal A-rich sequence and a 3′-end non-coding region. All these viruses also share *cis* acting signals at their 5′ and 3′ termini of the RNA positive strand for transcription and replication, respectively. However, they do not share a relevant overall sequence identity with either the full nucleotide sequence of dsRNA or their toxin amino-acid sequences (Ramírez et al., 2015, 2017).

The objective of this study was to determine the genome organization of the *T. delbrueckii* killer Kbarr1 strain LA virus as well as of other LA viruses from various *S. cerevisiae* killer strains isolated from the same geographical region. We addressed the following issues: (i) purification, sequencing, and characterization of TdV-LAbarr1, (ii) purification, sequencing, and characterization of several ScV-LA viruses from different types of *S. cerevisiae* killer strains, and (iii) analysis of the TdV-LAbarr1 genome organization and its Gag-Pol ORF as compared with the dsRNAs of other LA viruses. We discuss the evolutionary relationship between these yeast viruses, as well as the possible secondary structure and function of the 5′- and 3′-extra sequences found in the newly sequenced genomes.

## Materials and Methods

### Yeast Strains and Media

*Torulaspora delbrueckii* killer Kbarr1 yeast is a prototrophic strain isolated from the spontaneous fermentation of grapes from vines located in the Albarregas river valley in Spain (Ramírez et al., 2015). The industrial use of these Kbarr1 yeasts is under patent application. The *S. cerevisiae* killer strains EX231, EX1125, EX229, EX436, and EX1160 are also isolated from wine spontaneous fermentations in the Ribera del Guadiana region, which includes the Albarregas river valley, in Extremadura (southwestern Spain). We chose these strains because they present different mtDNA RFLP profiles and contain different isotypes of M dsRNA (Maqueda et al., 2010, 2012; Ramírez et al., 2017). All these yeasts are also prototrophic strains. The killer phenotype and the presence of viral dsRNA (L and M) in these yeasts have been analyzed previously (Rodríguez-Cousiño et al., 2011; Ramírez et al., 2015, 2017). The genomes of LA, LBC, and Mlus4 dsRNAs from EX229 have been analyzed by traditional techniques of cloning and sequencing (Rodríguez-Cousiño et al., 2011, 2013; Rodríguez-Cousiño and Esteban, 2017), and those of Mbarr1, M1-1, M2-4, Mlus1, Mlus4, and MlusA (from EX1180, EX231, EX1125, EX436, EX229, and EX1160, respectively) by HTS techniques (Ramírez et al., 2017). Table 1 summarizes the yeasts used in this study.

**TABLE 1:** Yeasts used in this study.

Strain	Genotype (relevant phenotype)	Origin, date, grape variety, geographical location in Extremadura (Spain)
*Td* EX1180	*wt* LAbarr1 LBCbarr1 Mbarr1 [Kbarr1^+^]	Wine, 2006, Cayetana, Albarregas (Mérida)
*Sc* EX231	*MAT a/*α *HO/HO* LA1 LBC M1-1 [K1^+^]	Wine, 2003, Macabeo, Guadajira
*Sc* EX1125	*MAT a/*α *HO/HO* LA2 LBC M2-4 [K2^+^]	Wine, 2005, Moscatel, La Albuera
*Sc* EX436	*MAT a/*α *HO/HO* LAlus1 Mlus1 [Klus^+^]	Wine, 2003, Tempranillo, Guadajira
*Sc* EX229	*MAT a/α HO/HO cyhS/cyhS* LAlus4 LBC Mlus4 [Klus^+^]	Wine, 2003, Macabeo, Guadajira
*Sc* EX1160	*MAT a/*α *HO/HO* LAlusA LBC MlusA MlusB MlusC [Klus^+^]	Wine, 2005, Moscatel, La Albuera

**Sc, Saccharomyces cerevisiae; Td, Torulaspora delbrueckii.**

Standard culture media were used for yeast growth (Guthrie and Fink, 1991). The YEPD contained 1% yeast extract, 2% peptone, and 2% glucose. The corresponding solid medium also contained 2% agar.

### Purification of dsRNA From LA Viruses

Samples containing total nucleic acids from killer yeast strains were obtained as described previously (Maqueda et al., 2010; Ramírez et al., 2015). Briefly, yeasts were placed in 10 mM Tris-HCl (pH 7.5) buffer containing 0.1 M NaCl, 10 mM EDTA, and 0.2% SDS. An equal volume of phenol (pH 8.0) was then added, and the mixtures incubated at room temperature for 30 min with shaking. Samples were centrifuged, and nucleic acids recovered in the aqueous phase were precipitated with isopropanol, washed with 70% ethanol, dried, and dissolved in TE buffer pH 8.0. The L and M dsRNAs were obtained from each yeast strain by CF-11 cellulose chromatography (Toh-e et al., 1978). After 1% agarose gel electrophoresis of each sample, the slower-moving dsRNA band (4.6 kb) was cut out of the gel and purified with RNaid Kit (MP Biomedicals, LLC, Illkrich, France). This procedure was repeated for each yeast strain to obtain at least 20 μg of each purified dsRNA.

### Preparation of cDNA Libraries From Purified V-L dsRNA and DNA Sequencing

The cDNA library preparation and high-throughput sequencing (HTS) were done at the Unidad de Genómica Cantoblanco (Fundación Parque Científico de Madrid, Spain) as has previously been described (Ramírez et al., 2017). Briefly, libraries from TdV-Lbarr1 (4.6 kb dsRNA purified band) were prepared with the “TruSeq RNA Sample Preparation kit” (Illumina) using 200 ng of purified dsRNA as input. The protocol was started at the fragmentation step, skipping the RNA purification step as the viral dsRNA had previously been purified. To facilitate the dsRNA denaturation, 15% DMSO was added to the Illumina fragment-prime solution before incubation at 94°C for 8 min. The first strand of cDNA was synthesized using random primers (dTVN and dABN oligonucleotides from Isogen Life Science, De Meern, The Netherlands) and SuperScriptIII retrotranscriptase. Then, the second cDNA strand synthesis, end repair, 3′-end adenylation, and ligation of the TruSeq adaptors were done (Illumina). These adaptor oligonucleotides include signals for further amplification and sequencing, and also short sequences referred to as indices which allow multiplexing in the sequencing run. An enrichment procedure based on PCR was then performed to amplify the library, ensuring that all the molecules in the library included the desired adaptors at both ends. The final libraries were denatured prior to seeding on a flow cell, and sequenced on a MiSeq instrument using 2 × 80 – 2 × 150 sequencing runs.

### dsRNA Sequence Assembly

The cDNA sequences obtained were analyzed and assembled by the firm Biotechvana (Technological Park of Valencia, Spain) basically as has previously been described (Ramírez et al., 2017). As a modification of this method, first, SOAP deNOVO2 (Luo et al., 2012) was used to obtain a *de novo* assembly based on two Illumina libraries for each virus, trying multiple assembly attempts with scaffolding and insert size of 200 and varying the Kmer value, with 47 found to be the most effective. This K47 assembly comprised several contigs and scaffolds. Contigs of size shorter than 300 nucleotides were removed from the contig file, while the remaining contigs were used as input to the NR database of the NCBI via the BLASTX search protocol (Altschul et al., 1997) implemented in the GPRO 1.1 software (Futami et al., 2011). Highly significant similarity was found between several contigs/scaffolds and some known viral RNA sequences (LA, LBC, and others) or host transcripts. Supposed contaminating sequences non-homologous to previously known LA genomes were filtered from the assembly. Each virus was sequenced at least three times using independent samples and different dates during a period of several years. Full coverage of the canonical genome sequence was obtained at least twice for each virus, and 100% identity was found between all sequences obtained from the same yeast strain. Coverage of the 5′ and 3′ extra sequences was 100% in at least two replicates from each virus. These extra 5′ and 3′ sequences were the same for each biological replicate. Only these full coverage sequences were considered for comparison of viral genomes from different yeasts.

### Miscellaneous

The sequence identity and phylogenetic relationship (phylogram) among LA genomes were obtained by the ClustalW(2.1) program for comparing nucleotide sequences (Thompson et al., 1994), and the MUSCLE(3.8) program for comparing amino-acid sequences (Madeira et al., 2019). The MFOLD program^1^ was used to predict the folding and hybridization of ssRNA (Zuker et al., 1999), and the FORNA program^2^ to visualize the RNA secondary structure (Kerpedjiev et al., 2015). The parameters used in MFOLD were: folding temperature fixed at 37°C; ionic conditions, 1 M NaCl, no divalent ions; percent suboptimality number, 5; upper bound on the number of computed foldings, 50; maximum interior/bulge loop size, 30; maximum asymmetry of an interior/bulge loop, 30; maximum distance between paired bases, no limit.

### Nucleotide Sequence Accession Numbers

The cDNA nucleotide sequence and amino-acid sequence of the Gag-Pol protein of newly sequenced (HTS) LA viruses appear in NCBI/GenBank under the following accession numbers: TdV-LAbarr1, (MW174763); ScV-LA1 from strain EX231, (MW174760); ScV-LA2 from EX1125, (MW174759); ScV-LAlus1 from EX436, (MW174761); and ScV-LAlusA from EX1160 (MW174762). These ScV-LA viruses are those described previously (Rodríguez-Cousiño et al., 2011; Ramírez et al., 2017) but *de novo* sequenced by HTS techniques for this study. The previously described ScV-L-A-lus from EX229 (Rodríguez-Cousiño et al., 2013) is here named ScV-LAlus4 because it comes from a killer Klus-4 type strain, and was *de novo* sequenced by HTS (accession number: MW174758). The genome sequences of other LA viruses used in this study are described in Table 2.

**TABLE 2:** Previously known sequence of yeast LA viruses used in this study.

Virus	Accession number	Reference/comment
ScV-LA1-original	J04692.1	Icho and Wickner, 1989. Previously known as ScV-LA. Renamed in this study to distinguish it from other LA-virus from different K1 killer yeasts
ScV-L-A-lus	JN819511.1	Rodríguez-Cousiño et al., 2013
ScV-LA2-8F13	KC677754.1	Rodríguez-Cousiño and Esteban, 2017
SpV-LA28	KU845301.2	Konovalovas et al., 2016. Formerly assigned to S. cerevisiae but recently re-assigned to *S. paradoxus* (Vepštaitë-Monstavičė et al., 2018)
SpV-LA21	KY489962.1	Rodríguez-Cousiño et al., 2017
SpV-LA45	KY489963.1	Rodríguez-Cousiño et al., 2017
SpV-LA74	KY489964.1	Rodríguez-Cousiño et al., 2017
SpV-LA4650	KY489965.1	Rodríguez-Cousiño et al., 2017
SpV-LA1939	KY489966.1	Rodríguez-Cousiño et al., 2017
SpV-LA1143	KY489967.1	Rodríguez-Cousiño et al., 2017
SpV-LA62	KY489968.1	Rodríguez-Cousiño et al., 2017
SpV-LA66	MH784501.1	Vepštaitë-Monstavičė et al., 2018
SkV-LA1082	KY489970.1	Rodríguez-Cousiño et al., 2017
SkV-LAFM1183	KX601068.1	Rowley et al., 2016
SuV-LA10560	KY489969.1	Rodríguez-Cousiño et al., 2017

**Sc, S. cerevisiae; Sp., S. paradoxus; Sk, S. kudriavzevii; Su, S. uvarum.**

## Results

### Analysis of the dsRNA and Gag-Pol Sequences From TdV-LAbarr1

**FIGURE 1:**
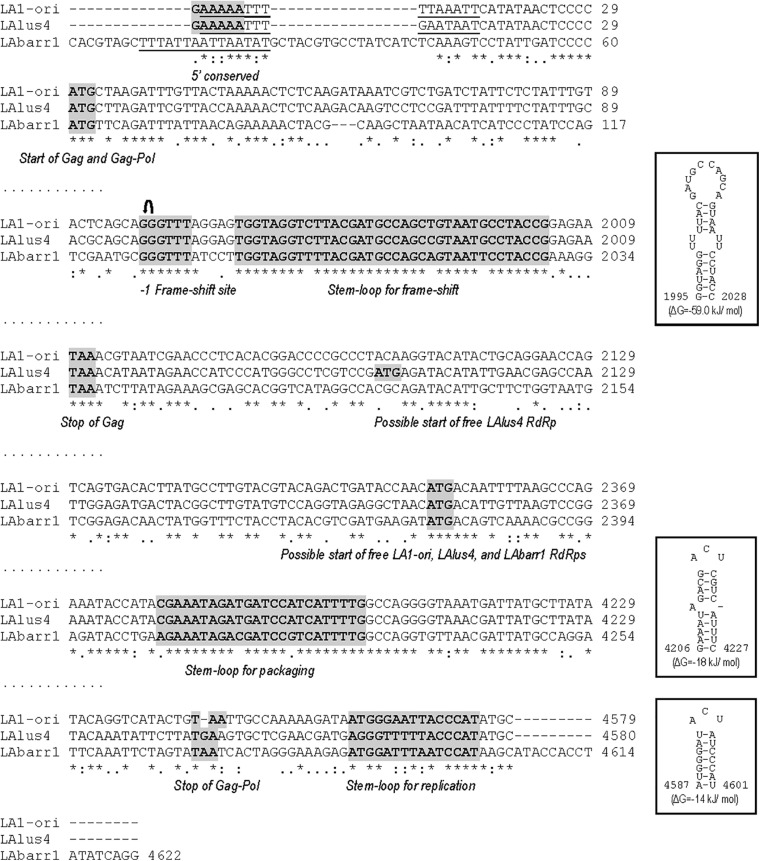
Partial multiple sequence alignment between ScV-LA-original, ScV-LAlus4, and TdV-LAbarr1 (+) strand nucleotide sequences (cDNA). The full sequence alignment is presented in Supplementary Figure S1. 5′GAAAAA conserved motif (5′ conserved), translation initiation (start of Gag and Gag-Pol, or internal ATG in Pol ORF of ScV-LA-original, ScV-LAlus4, and TdV-LAbarr1) and termination (stop of Gag or Gag-Pol) codons, ribosome frameshifting site (−1 frameshift site), frameshifting associated sequence (stem loop for frameshift), packaging signal (stem loop for packaging), and replication signal (stem loop for replication) are indicated and gray shaded in the nucleotide sequence. An AU-rich 15-nucleotide stretch located in the 5′ untranslated terminal region is underlined.↶, ribosomal frameshift. Asterisks (*), colons (:), and dots (.) indicate identical nucleotide positions, transitions, and transversions, respectively. The secondary structures of the putative *cis* signals for frameshifting, packaging, and replication of TdV-LAbarr1 are displayed at the right of the sequence panel.

Two different sequences were obtained from the L band (4.6 kb) cDNA present in the Kbarr1 strain—one shows above 60% nucleotide identity with the ScV-LA-original genome (that we named TdV-LAbarr1), and the other shows above 56% nucleotide identity with the ScV-LBC genome (TdV-LBCbarr1). The full sequence obtained for TdV-LAbarr1 cDNA is of 4,622 nucleotides, which is very close to the size estimated by agarose-gel electrophoresis. Most of this sequence (4,591 nt central stretch) shows 62% nucleotide identity with previously known ScV-LA-original and ScV-LAlus4 (Supplementary Figures S1, S3), while these two ScV-LA genomes share a greater identity of 74% (Rodríguez-Cousiño et al., 2013). This central stretch is therefore considered to be the canonical sequence of the TdV-LAbarr1 genome, and sequences upstream (14 nt) and downstream (17 nt) as 5′- and 3′-extra sequences, respectively (Figure 1). The TdV-LAbarr1 genome organization is quite similar to that of ScV-LA-original and ScV-LAlus4, with the three viral RNAs containing two ORFs. Based on the sequence homology, the first TdV-LAbarr1 sequence ORF (from nt 61 to 2,093) can be assigned as being the coat (Gag) protein of the virion, and the second ORF (from nt 2,376 to 4,567) as the viral RNA-dependent RNA polymerase (RdRp). This polymerase is probably expressed as a Gag-Pol fusion protein together with Gag ORF by a -1 ribosomal frameshift at the conserved frameshifting site located upstream of the Gag ORF stop codon (from nt 1,983 to 1,988) (Figure 1 and Supplementary Figure S1). These ORF assignments are also based on the amino-acid sequence homology of the Gag-Pol fusion protein of TdV-LAbarr1 to those of ScV-LA-original and ScV-LAlus4 (see below). Nonetheless, these three LA genomes have one or two putative in-frame translation re-initiation start codons downstream of the Gag-ORF stop codon and upstream of the Pol domain (indicated in boldface in Figure 1 and Supplementary Figure S1).

Furthermore, as described previously for ScV-LA-original and ScV-LAlus4 (Rodríguez-Cousiño et al., 2013), there are three regions in the TdV-LAbarr1 genome that are highly conserved: (i) the stem-loop region known to be involved in frameshifting (nt 1,969 to nt 2,004) adjacent to the slippery site 1983GGGUUUA1989 in TdV-LAbarr1, (ii) a 24 nt stem-loop region responsible for binding to and packaging of the LA (+) strand (nt 4,205 to nt 4,228 in TdV-LAbarr1), and (iii) a 15-nt stem-loop region responsible for RNA replication (nt 4,587–4,601 in TdV-LAbarr1) (Figure 1). This indicates the importance of these regions in the translation, packaging, and replication steps of these viruses. Only three nucleotide changes are found in the packaging stem loop of TdV-LAbarr1 with respect to ScV-LA-original or ScV-LAlus4 (C4180A, T4189C, and A4195G), and these changes even slightly decrease the free energy of the structure from ΔG = -16 kJ/mol in ScV-LA-original to ΔG = -18 kJ/mol in TdV-LAbarr1. The frameshifting stem loop of TdV-LAbarr1 is also highly conserved with respect to those of ScV-LA-original and ScV-LAlus4. Only three nucleotide changes are found (C1977T and G1997T with respect to ScV-LA-original and ScV-LAlus4, and T1991A with respect to ScV-LA-original or C1991A with respect to ScV-LAlus4), which slightly increase the free energy of the structure from ΔG = -64 kJ/mol in ScV-LA-original to ΔG = -59 kJ/mol in TdV-LAbarr1. Some other nucleotide changes are also found in the replication stem-loop sequence of TdV-LAbarr1 with respect to ScV-LA-original (G4565A, A4566T, A4567T, T4569A, and C4571T) and ScV-LAlus4 (G4562T, T4565A, T4569A, and C4571T). These changes do not alter the putative stem-loop structure, although they increase the free energy of the structure from ΔG = -21 kJ/mol in ScV-LA-original to ΔG = -14 kJ/mol in TdV-LAbarr1. Nonetheless, it has previously been described that the nucleotide sequence of the loop (here conserved) is important but that of the stem is not (Esteban et al., 1989; Rodríguez-Cousiño et al., 2013).

**FIGURE 2:**
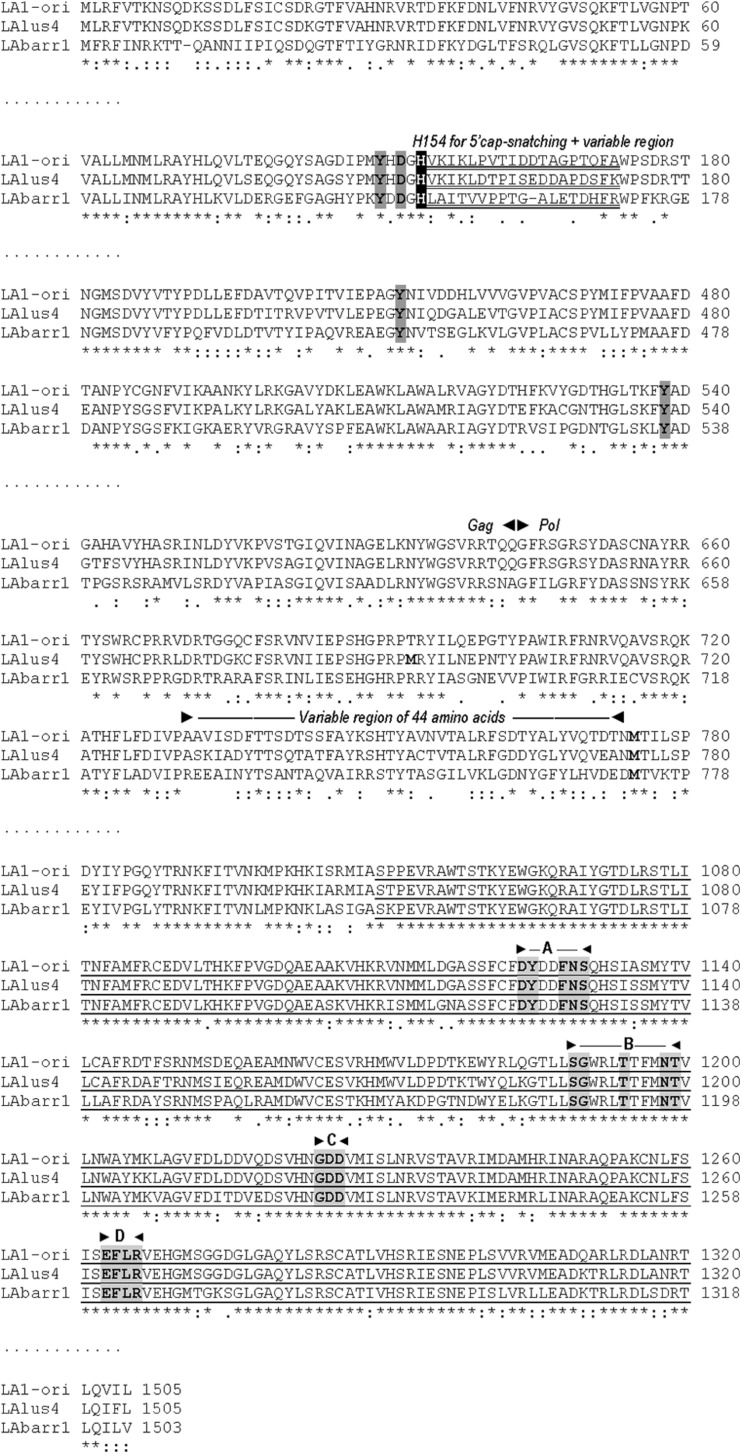
Comparison between partial amino-acid sequences of Gag-Pol encoded by ScV-LA-original, ScV-LAlus4, and TdV-LAbarr1 dsRNA genomes. The full sequence alignment is presented in Supplementary Figure S2. The separation between Gag and Pol is indicated (Gag◀▶Pol). The H154 residue required for 5′cap-snatching is black shaded. The stretch of variable amino-acid sequence located downstream from H154 is double underlined. The four crucial residues for cap recognition (Tyr-150, Asp-152, Tyr-452, and Tyr-538) are gray shaded. A variable region of 44 amino acids in the N-terminal region of Pol is indicated above the sequence. The highly conserved central third of Pol is underlined, and the four consensus motifs (A–D) conserved in RNA-dependent RNA polymerases from totiviruses are indicated above the sequence, and the conserved amino acids for each motif are gray shaded. Methionines (M) in the N-terminal region of Pol are in boldface. Asterisks (*) indicate identical amino acids; colons (:) and single dots (.) indicate conserved and semi-conserved amino acids, respectively.

Despite the aforementioned similarities, TdV-LAbarr1 differs from the ScV-LA-original and ScV-LAlus4 genomes in that it contains: (i) 14 extra nucleotides at the 5′ end, (ii) 17 extra nucleotides at the 3′ end, (iii) a 17-nucleotide non-homologous stretch close to the 5′ end, and (iv) it does not have the conserved 5′GAAAAA motif present in ScV-LA-original and ScV-LAlus4 (Figure 1 and Supplementary Figure S1). Although no experimental evidence has been reported, it has been suggested that this conserved motif is related to the supposed *cis* signals required for transcription, similarly to other 5′ AU-rich regions in dsRNA viruses that facilitate the “melting” of the molecule and the access of the RNA polymerase to the template strand for conservative transcription (Rodríguez-Cousiño et al., 2013). In this sense, the 5′ UTR (untranslated terminal region) of the three LA viruses contains an AU-rich 15-nucleotide stretch (100% AU for ScV-LA-original, 93.3% for ScV-LAlus4, and 100% for TdV-LAbarr1) that may be responsible for facilitating this melting. The putative Gag-Pol amino-acid sequence of TdV-LAbarr1 shows 62 and 63% identity with that of ScV-LA1-original and ScV-LAlus4, respectively, while those of ScV-LA1-original and ScV-LAlus4 share a greater identity of 87% (Rodríguez-Cousiño et al., 2013; Figure 2 and Supplementary Figure S2). The identity shared by these three Gag-Pol proteins is good enough to expect similar spatial organization and polymerase functional behavior for all these LA viruses. The Gag His154 residue (His153 in TdV-LAbarr1) required for the cap-snatching mechanism (transferring cap groups from other yeast mRNAs to the nascent mRNA of LA virus when extruded from the virion; Fujimura and Esteban, 2011) in *S. cerevisiae* viruses and the four crucial residues for 5′cap recognition (Tyr-150, Asp-152, Tyr-452, and Tyr-538 Fujimura and Esteban, 2013) are present in the Gag protein of the three viruses. Moreover, the central third of the Pol sequence, which is highly conserved among the RdRps (underlined in Figure 2 and Supplementary Figure S2), shares 82% identity with that of ScV-LA1-original, and the four conserved motifs in this region (A, B, C, and D; Bruenn, 2003) are 100% identical in the three Pol proteins. Other parts of Gag and Pol are also highly conserved. Worthy of mention among the poorly conserved regions are a 19-amino-acid variable region located downstream of H154/153, and the 44-amino-acid variable region located in the N-terminal third of Pol (amino acids 729A to 772D in ScV-LA-original Gag-Pol) in which only eight amino acids are identical (Figure 2), both of which stretches have previously been described as variable regions (Rodríguez-Cousiño et al., 2013).

### Comparison of TdV-LAbarr1 With LA Viruses From *Saccharomyces* Yeasts

The dsRNA and Gag-Pol sequences of TdV-LAbarr1 were compared with their counterparts from *Saccharomyces* yeasts to analyze their phylogeny. The sequences of *S. cerevisiae* ScV-LA1-original (Icho and Wickner, 1989), ScV-LAlus4 (Rodríguez-Cousiño et al., 2013), ScV-LA2-8F13 (Rodríguez-Cousiño and Esteban, 2017), and SpV-LA28 (Konovalovas et al., 2016) were already known, as also were the sequences of *S. paradoxus* SpV-LA21, SpV-LA45, SpV-LA74, SpV-LA4650, SpV-LA1939, SpV-LA1143, and SpV-LA62 (Rodríguez-Cousiño et al., 2017), *S. paradoxus* SpV-LA66 (Vepštaitë-Monstavičė et al., 2018), *S. kudriavzevii* SkV-LA1082 (Rodríguez-Cousiño et al., 2017) and SkV-LAFM1183 (Rowley et al., 2016), and *S. uvarum* SuV-LA10560 (Rodríguez-Cousiño et al., 2017). The viruses ScV-LA1 from strain EX231, ScV-LA2 from EX1125, ScV-LAlus1 from EX436, and ScV-LAlusA from EX1160 have been described previously (Rodríguez-Cousiño et al., 2011; Ramírez et al., 2017), and their genomes were *de novo* sequenced by HTS techniques for this study. ScV-LAlus4 from EX229, previously named ScV-L-A-lus (Rodríguez-Cousiño et al., 2013), was also de novo sequenced as a control to assess the accuracy of our HTS procedure. Only two nucleotide changes were found in the nucleotide sequence of ScV-LAlus4 with respect to ScV-L-A-lus: G2434A and A3645T. As nucleotides of ScV-LAlus4 (HTS) in these two positions coincided best with the rest of the *S. cerevisiae* LA viruses, this sequence was the one used for further analyses.

**FIGURE 3:**
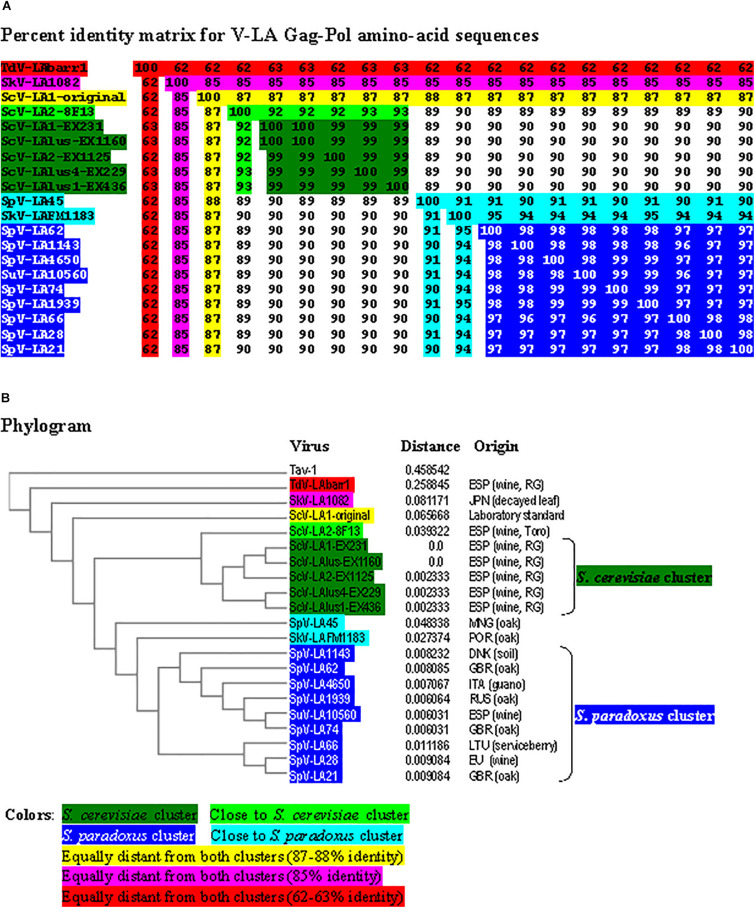
Phylogenetic relationship of yeast LA viruses. **(A)** Percentage identity matrix between the complete amino acid sequences of the Gag-Pol proteins of LA viruses. Each identity value is rounded to the nearest whole number. **(B)** Phylogram with evolutionary distances (given by the MUSCLE program) and geographical location at which each killer yeast strain was isolated. RG, Ribera del Guadiana. Country codes are in accordance with the International Organization for Standardization ISO 3166-2. Viruses included in each of the two main clusters, as well as their identity values, are shaded in dark-green or dark-blue. The relative identity with these clusters of the rest of the viruses is indicated by shading in other colors.

The percentage of identity among the different viruses was always greater for Gag-Pol amino-acid sequences than for genomic nucleotide sequences (Figure 3 and Supplementary Figure S3), similar to earlier findings for *Saccharomyces* LA viruses (Rodríguez-Cousiño et al., 2017; Vepštaitë-Monstavičė et al., 2018). Two main clusters were found to include most LA virus sequences: a *S. cerevisiae* cluster that grouped the viruses of all the wine yeasts isolated from the Region of Extremadura (99.2–100% identity of Gag-Pol), and a *S. paradoxus* cluster that grouped most viruses of this yeast species (except for SpV-LA-45) and SuV-LA10560 of *S. uvarum* (96.3–98.8% identity) (Figure 3 and Supplementary Figure S3).

Viruses SpV-LA45 of *S. paradoxus* and SkV-LAFM1183 of *S. kudriavzevii* were closer to the *S. paradoxus* than the *S. cerevisiae* cluster (90–95% and 89–90% identity of Gag-Pol, respectively), and both viruses show 91% identity in their Gag-Pol amino-acid sequence. The virus ScV-LA2-8F13 of *S. cerevisiae* was closer to the *S. cerevisiae* than the *S. paradoxus* cluster (92–93% and 89–90% identity, respectively). The viruses SkV-LA1082 of *S. kudriavzevii* and ScV-LA1-original of *S. cerevisiae* were equally distant from both main clusters (85 and 87% identity, respectively), and both viruses show 85% identity in their Gag-Pol amino-acid sequence. Finally, the virus TdV-LAbarr1 of *T. delbrueckii* (isolated from Ribera del Guadiana, Extremadura, Spain) was the most distant from the rest of the viruses (62–63% identity) (Figure 3). Similar results were found for cluster formation and virus association when comparing separately the amino-acid sequence of Gag and the highly conserved RdRp-domain of Pol. However, the percentage identity between RdRp domains was greater than with comparisons using full Gag-Pol sequences (compare Figure 3A and Supplementary Figure S4). On the contrary, the percentage identity between Gag domains was less than with comparisons using full Gag-Pol sequences, and ScV-LA1-original was then slightly closer to the *S. cerevisiae* (80–81%) than to the *S. paradoxus* cluster (74–78%) (compare Figure 3A and Supplementary Figure S5). The greatest change was found for TdV-LAbarr1. The TdV-LAbarr1 Gag sequence was approximately 18% lower in identity (from 62–63% to 44–47%), while the TdV-LAbarr1 RdRp domain sequence was approximately 22% higher (from 62–63% to 82–85%), in both cases with respect to the rest of the viruses (compare Figure 3A and Supplementary Figures S4, S5). Thus, Gag amino-acid sequence comparison seems to be the most efficacious procedure for grouping all the viruses from each yeast species. As was to be expected, identity between most viruses decreased when comparing the variable hydrophobic 44-amino-acid stretch located in the N-terminal region of Pol. The exception was among viruses of the *S. cerevisiae* cluster that increased up to 100% identity in all cases (compare Figure 3A and Supplementary Figure S6). The results were similar in comparing the variable 19-amino-acid stretch located downstream of H153/154 of Gag (compare Figure 3A and Supplementary Figure S7). This was not found, however, for viruses of the *S. paradoxus* cluster in which the identity values for these variable stretches decreased, probably because these yeast strains were isolated from geographical locations that were insufficiently close.

### Analysis of the 5′- and 3′-Extra Sequences Found in TdV-LAbarr1 and Some ScV-LA Genomes

**FIGURE 4:**
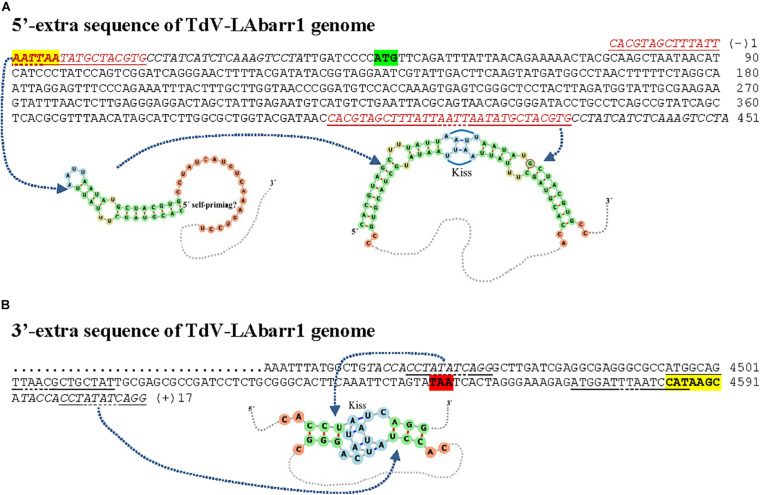
cDNA of 5′-extra **(A)** and 3′-extra **(B)** sequences, and proximal canonical sequences of TdV-LAbarr1 genome from *T. delbrueckii* EX1180. The 5′-AATTAA and CATAAGC-3′ ends of canonical sequences are in boldface and yellow highlighted. The protein synthesis initiation and stop codons of Gag-Pol are shaded in green and red, respectively. Nucleotides of palindromic sequences are shown in red. Duplicated sequences in each virus are in italics. Stem loops are underlined, and unpaired nucleotides of each loop are dot underlined. The secondary RNA structure of possible 5′ stem loops and kissing stem loops are shown at the bottom of the sequence.

**FIGURE 5:**
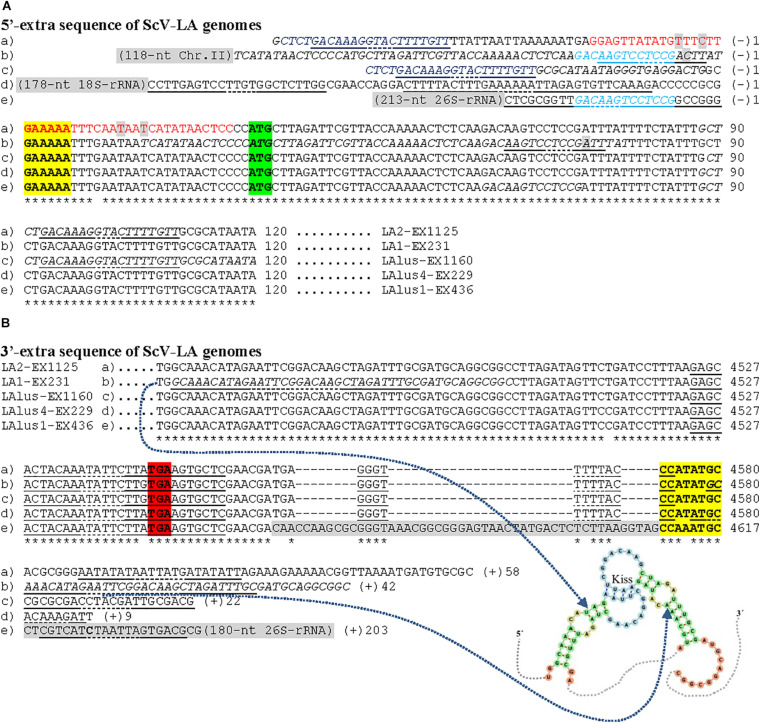
Nucleotide sequence (cDNA) alignment of 5′- **(A)** and 3′-extra **(B)** sequences, and proximal canonical sequences of ScV-LA genomes from *S. cerevisiae* strains isolated from Ribera del Guadiana: (a) LA2-EX1125; (b) LA1-EX231; (c) LAlusA-EX1160; (d) LAlus4-NGS; (e) LAlus1-EX436. Asterisks (*) indicate identical nucleotides. The 5′-GAAAAA and CCA(T/A)TGC-3′ ends of canonical sequences are in boldface and yellow highlighted. The protein synthesis initiation and stop codons of Gag-Pol are shaded in green and red, respectively. Sequence stretches that are homologous in two different virus genomes are light-blue or dark-blue shaded. The 5′-extra sequences that are homologous to rRNA or chromosome II sequences of *S. cerevisiae* are light- gray shaded. Nucleotides of palindromic sequences are shown in red. Duplicated sequences in each virus are in italics. Stem loops are underlined, and unpaired nucleotides of each loop are dot underlined. The possible kissing stem-loop secondary RNA structure in ScV-LA1-EX231 is shown at the bottom of the sequence.

The complete sequences obtained for the TdV-LAbarr1 and the five ScV-LA viruses from Ribera del Guadiana were longer than the former’s estimated canonical sequence or the latter’s previously known sequences (Table 3). Extra nucleotides were found on both sides of the canonical sequence—the 5′ and 3′ ends. For sequence descriptions in this section, nucleotides are numbered from the 5′GAAAAA conserved motif in ScV-LA viruses, which is generally accepted as the 5′-end in most *S. cerevisiae* viral L canonical genomes (Fujimura and Wickner, 1989). The homologous motif 5′AATTAA is considered for TdV-LAbarr1. The 5′-terminal G or A is denoted as number 1. Extra nucleotides found upstream from 5′GAAAAA or 5′AATTAA motif are numbered with a negative symbol starting at (−)1 from the first nucleotide upstream from 5′G or 5′A. Similarly, extra nucleotides found downstream from the previously considered 3′-end of ScV-LA genomes (CCATATGC3′, or CCAAATGC3′ in ScV-LAlus1 from the EX436 strain) and now considered for TdV-LAbarr1 (CCATAAGC3′) are numbered with a positive symbol starting at (+)1 from the first nucleotide located downstream from C3′ (Figures 4, 5).

**TABLE 3:** Characteristics of dsRNA LA-virus genomes sequenced by HTS.

Virus	Previous sequenced length (bp)/yeast strain	Newly analyzed yeast strain	Killer phenotype/M dsRNA isotype	Sequenced length in (bp) /canonical	5′-extra sequence (bp)/% identity to, size (position)	3′-extra sequence (bp)/% identity to, size (position)	Stem-loop involving 5′-extra sequence (position) (**Δ** G)	Stem-loop involving 3′-extra sequence (position) (**Δ** G)
LAbarr1	Unknown**/***Td* EX1180	*Td* EX1180	Kbarr1**/**Mbarr1	4622**/**4591	14**/**100% self-identity to (+) strand, 51 nt [C(−)14–A37] to 51 nt [C401 to A451]	17**/**100% self-identity to (+) strand, 16 nt [T(+)2–G(+)17] to 16 nt [T4457–G4472]	[C(−)14 to G(+)17] (−50)	[C(+)7 to G(+)17] (−2.5)
LA1-original	4579**/***Sc* RE59	*Sc* EX231	K1**/**M1-1	4874**/**4580	252**/**100% *Sc* chromosome II, 118 nt, [C(+)252–A(+)65]; and 100% self-identity to (+) strand, 58 nt [T(−)63–A(−)6] to 58 nt [T16 to A73]	42**/**100% self-identity to (+) strand, 44 nt [G4579–C(+)42] to 44 nt [G4457–C4500]	[A(−)16 to T(−)3] (−12)	[G4579 to C(+)30] (−24)
LA2	4580**/***Sc* 8F13	*Sc* EX1125	K2**/**M2–4	4696**/**4580	58**/**100% self-identity to (+) strand, 23 nt [G(−)58–T (−)36] to 23 nt [G88–T110]. Same as in LAlusEX1160	58**/**no identity found	[G(−)53 to U(−)36] (−15). Same as in LA-lus EX1160	[A(+)8 to U(+)28] (−6.3)
LAlus4	4580**/***Sc* S3920 = EX229	*Sc* EX436	Klus**/**Mlus1	5052**/**4617	232**/**93% identity to *Sc* 26S (−) rRNA, 213 nt [C(−)232 to T(−)20]	203**/**261 nt are 100% to *Sc* 26S (+) rRNA, [C4560 to T(+)203]	[C(−)28 to G(−)1] (−29)	[C(+)3 to G(+)21] (−20)
		*Sc* EX229	Klus**/**Mlus4	4776**/**4580	187**/**100% identity to *Sc* 18S (+) rRNA, 178 nt [G(−)187–G10]	9**/**no identity found	[C(−)74 to G(−)52] (−31), and [C(−)41 to G(−)10] (−16)	[A4577 to U(+)8] (−18)
		*Sc* EX1160	Klus**/**MlusA	4648**/**4580	46**/**100% self-identity to (+) strand, 44 nt [C(−)46–G(−)3] to 44 nt [C89–G132]. Same as in LA2-EX1125	22**/**no identity found	[G(−)42 to U(−)25] (−15). Same as in LA2-EX1125	[C(+)2 to G(+)22] (−20)

**ΔG was obtained for the ssRNA(+) with the program MFOLD. ΔG is in kJ/mol. Sc, Saccharomyces cerevisiae; Td, Torulaspora delbrueckii; the cDNA sequence was considered for nucleotide position.**

No relevant identity was found between the extra 5′ or 3′ sequences of the different LA viruses, or between these two extra sequences of each virus. However, 100% local identity was found among some stretches in the 5′-extra sequence of some *S. cerevisiae* LA genomes: 13 nt [5′-GACAAGTCCTCCG-3′] [G(−)7 to G(−)19] in ScV-LA1-EX231 and ScV-LAlus1-EX436, and 22 nt [5′-CTCTGACAAAGGTACTTTTGTT-3′] in ScV-LA2-EX1125 [C(−)57 to T(−)36] and ScV-LAlusA-EX1160 [C(−)47 to T(−)25] (Figure 5A).

A 51 nt stretch of TdV-LAbarr1, part in the 5′-extra sequence (14 nt) and part in the 5′-end (37 nt) [C(−)14 to A37], showed 100% identity with a homologous stretch located near the 5′-end in the canonical sequence of the same virus [C401 to A451] (viral self-identity). This stretch is almost a palindromic sequence, and about half of this sequence (5′-CACGTAGCTTTATTAATTAATATGCTACGTG-3′) can form a stem loop (ΔG = -50 kJ/mol), a loop that contains the first four nucleotides of the canonical sequence (5′-AATT) as unpaired. These nucleotides are complementary to the unpaired nucleotides in the stem loop of the homologous stretch located downstream in the RNA sequence (5′-AAUU/3′-UUAA), and it is possible that a kissing complex could be formed by four-base-pair annealing (Figure 4A). A similar palindromic sequence (44 nt), with the 5′GAAAAATTT conserved motif included at about the middle of the stretch, capable of forming a stem loop (ΔG = -71 kJ/mol) was also found in ScV-LA2-EX1125. Additionally, a stretch in the 5′-extra sequence that has 100% identity with a homologous stretch located close to the 5′GAAAAA conserved motif in the canonical sequence was also found in the same ScV-LA2-EX1125 virus (viral self-identity). This conserved motif was similar to that also found in the rest of the ScV-LA viruses except for ScV-LAlus4-EX229. However, ScV-LAlus4-EX229 showed two stem loops in the 5′-extra sequence. Another stem loop, close to the 5′GAAAAATTT conserved motif in the canonical sequence, was also found in all ScV-LA viruses (Figure 5A). Nevertheless, no probable kissing-loop interactions were found near the 5′-end of *S. cerevisiae* viruses. Other interesting sequences were also found in ScV-LA1-EX231, ScV-LAlus4-EX229, and ScV-LAlus1-EX436: 118 nt of 100% identity with a chromosome II sequence of *S. cerevisiae*, 178 nt of 100% identity with *S. cerevisiae* 18S rRNA, and 213 nt of 93% identity with *S. cerevisiae* 26S rRNA, respectively (Table 3 and Figure 5A).

With respect to 3′-extra sequences, a 16 nt stretch [T(+)2 to G(+)17] of TdV-LAbarr1 showed 100% identity with a homologous stretch located close to 3′-extra in the canonical sequence of the same virus [T4457 to G4472], and part of each can form a stem loop (ΔG = -2.5 kJ/mol). The unpaired nucleotides of these homologous RNA loops are complementary to each other (5′-AUAU/3′-UAUA). Therefore, similarly to the case for the 5′-end of the same virus, intramolecular kissing-loop interaction is possible through four-base-pair annealing (Figure 4B). A similar situation was found in ScV-LA1-EX231, whose 3′-extra sequence contains a 44 nt stretch [G4579 to C(+)42] of 100% identity with a homologous stretch located close to the 3′-end of the canonical sequence of the same virus [G4457 to C4500], which can also form a stem loop (ΔG = -24 kJ/mol) with unpaired nucleotides compatible with a kissing RNA interaction (5′-AAUU/3′-UUAA). No stretch with viral self-identity was found in 3′-extra sequences of ScV-LA2-EX1125, ScV-LAlus1-EX436, ScV-LAlus4-EX229, or ScV-LAlusA-EX1160, but a possible stem loop was found in every case, and one or two stem loops were also found close to the 3′-end of the canonical sequence of each virus (Figure 5B). No kissing-loop interaction was detected, however, for any of these four viruses. Further additional sequences were also found in ScV-LAlus1-EX436, 261 nt [C4560 to T(+)203] with 100% identity with *S. cerevisiae* 26S rRNA (Table 3 and Figure 5B), which could also be involved in kissing-like interactions. This sequence stretch belongs to a part of the 26S-rRNA different from that found in the 5′-extra sequence of the same virus.

## Discussion

### Analysis of the TdV-LAbarr1

The average nucleotide similarity of TdV-LAbarr1 with ScV-LA1-original and ScV-LAlus4 was high, but not as high as that found between both *S. cerevisiae* viruses. This was to be expected given that they come from different yeast species with different ecological niches—*S. cerevisiae* is rarely isolated from natural environments (Kurtzman, 2011a), while *T. delbrueckii* in widely distributed in nature (Kurtzman, 2011b)—and no external infection capability has yet been described for these viruses. However, the TdV-LAbarr1 genome organization is quite similar to that of ScV-LA-original and ScV-LAlus4. It contains the same two Gag and Pol ORFs and shares 87.5–100% identity in some regions considered important for the virus replication cycle, such as the frameshifting region that facilitates the fusion of Gag and Pol or the virus packaging signal. Nevertheless, the few nucleotide changes we found in the frameshifting region may affect the frameshift efficiency, and hence the ratio between Gag and Gag-Pol (Dinman and Wickner, 1992). With respect to the 5′ and 3′ untranslated terminal regions, where important *cis* signals for transcription and replication are located, the degree of sequence conservation observed was less than that of the translated region—only 67% conservation in the 3′-end replication stem-loop sequence and absence of the conserved 5′GAAAAA motif in TdV-LAbarr1. Despite this, as has previously been argued (Rodríguez-Cousiño et al., 2013), it is likely that the secondary or tertiary structure of the 3′-end replication stem loop is the feature required for LA virus replication instead of the sequence itself. Similarly, despite the absence of the 5′GAAAAA motif in TdV-LAbarr1, it does contain an AU-rich 15-nucleotide stretch (100% AU for ScV-LA-original and TdV-LAbarr1) that could be responsible for facilitating the melting of the molecule and the access of the RNA polymerase to the template strand for the conservative transcription (Rodríguez-Cousiño et al., 2013). Additionally, 14 extra nucleotides at the 5′ end, 17 extra nucleotides at the 3′ end, and a 17-nucleotide non-homologous stretch close to the 5′ end account for the rest of the relevant differences of TdV-LAbarr1 with ScV-LA-original and ScV-LAlus4. As the ends of these dsRNA genomes are usually harder to sequence accurately than the rest of the molecule, the finding of these extra sequences suggests that they might have been missed in previously published ScV-LA-original and ScV-LAlus4 genomes that were sequenced using traditional cDNA sub-cloning approaches (Urayama et al., 2018, 2020). Such a missing sequence might be the reason for the failure up to now of LA launching experiments between different yeast strains (Valle and Wickner, 1993).

In agreement with the nucleotide sequence, the amino-acid sequence identity of TdV-LAbarr1 Gag-Pol with that of ScV-LA-original and ScV-LAlus4, while high, is lower than that between ScV-LA-original and ScV-LAlus4. Once again, however, the relevant features previously described for the ScV-LA-original Gag and Pol proteins (Rodríguez-Cousiño et al., 2013) are conserved in TdV-LAbarr1. In particular, these are as follows: a similar central part of Gag, probably reflecting structural constraints of Gag to interact with another Gag subunit and form the asymmetric Gag dimer present in the icosahedral LA virion; the 100% identity of the His154 residue (His153 in TdV-LAbarr1) required for cap-snatching and the four crucial residues for cap recognition (Blanc et al., 1994; Tang et al., 2005); and the four conserved motifs in the central domain of the RdRps (Ribas and Wickner, 1992; Bruenn, 2003). Even two non-homologous regions that can be considered as relevant Gag-Pol features, which should not require a high degree of conservation among the different Gag-Pol fusion proteins, are also present. These are the 19-amino-acid variable stretch located downstream from the aforementioned His-153 (likely to be facing the outer surface of the virion and probably not structurally important), and the hydrophobic 44-amino-acid variable stretch located in the N-terminal region of Pol (likely to be separating the Gag and Pol domains in the fusion protein) (Rodríguez-Cousiño et al., 2013). All these features, similar to those already found in *S. cerevisiae*, point to TdV-LAbarr1 being a typical LA virus sharing a lower sequence identity than ScV-LA-original and ScV-LAlus4 because it belongs to a different yeast genus.

### Phylogenetic Relationship of TdV-LAbarr1 and LA Viruses From *Saccharomyces* Yeasts

The phylogenetic relationship we found for *Saccharomyces* LA viruses is similar to that reported previously (Vepštaitë-Monstavičė et al., 2018), showing two main clusters, one including mainly *S. cerevisiae* viruses, and another including most *S. paradoxus* viruses. An alternative proposal is of two clades for LA viruses included in the *S. paradoxus* cluster: an LA-28 type, including SpV-LA-66, SpV-LA-21, and SpV-LA-28, and the rest of the *S. paradoxus* viruses (Rodríguez-Cousiño et al., 2017; Vepštaitë-Monstavičė et al., 2018). However, contrary to what has previously been described (Vepštaitë-Monstavičė et al., 2018), LA viruses from *S. cerevisiae* seem to be more homogeneous than those from *S. paradoxus*. This may just be a reflection of the geographical closeness of the locations at which the *S. cerevisiae* strains were collected from which the new LA viruses included in our study were isolated. Further analysis of new killer yeasts isolated from different, well documented, geographical locations (close and distant) is needed to clarify this issue.

Comparing the amino acid sequence of Gag proteins represents an interesting approach to study the co-evolution of each LA virus with each yeast species. Indeed, we found that this approach was clearly the best method to group all yeast strains of the same species, at least in the case of *S. cerevisiae*. Surprisingly, two stretches of the Gag-Pol sequences previously known as poorly conserved (Rodríguez-Cousiño et al., 2013) were those most conserved among the *S. cerevisiae* viruses isolated from the same geographical region (spontaneous wine fermentation, Ribera del Guadiana, Spain), regardless of which type of M killer virus was supported by these helper LA viruses in each case. However, the opposite was found when comparing the same sequence stretches of the viruses included in the *S. cerevisiae* cluster with the rest of the viruses. The corresponding identity percentage was the lowest found even when the viruses belonged to the same yeast species isolated at only 300 km distance (K2 *S. cerevisiae* 8F13 from Toro and *S. cerevisiae-*cluster yeasts from Ribera del Guadiana, both in Spain), to different yeast species isolated in the same area (Kbarr1 *T. delbrueckii* EX1180 and *S. cerevisiae*-cluster yeasts, all from Ribera del Guadiana, Spain), or to different yeast species isolated at only 120 km distance (*S. kudriavzevii* FM1183 from Castelo de Vide, Portugal and *S. cerevisiae-*cluster yeasts from Ribera del Guadiana, Spain). In particular therefore, if 100% identity is found in these two variable sequence stretches of Gag-Pol, those LA viruses may be considered to come from the same yeast species isolated from the same geographical area, no matter which type of M killer virus coincides with the LA virus in the same yeast strain. Therefore, these LA-virus variable sequences could be a good genetic marker of the unknown geographical origin of a given yeast killer strain.

Contrary to a previous suggestion (Rodríguez-Cousiño and Esteban, 2017; Rodríguez-Cousiño et al., 2017), there seems to occur no specific association of each toxin-producing M with its helper virus as a result of viral co-evolution. Therefore, as dsRNA nucleotide and Gag-Pol amino-acid sequence identity of ScV-LA viruses depends mostly on the geographical location at which the *S. cerevisiae* strains were isolated, viral cross transmission between yeast strains of the same species living in the same habitat is to be expected. The lowest identity among ScV-LA viruses was found between different yeast species, even if they came from the same geographical location, as was the case for *T. delbrueckii* EX1180 and the *S. cerevisiae*-cluster strains. Therefore, contrary to what has previously been suggested for some yeast species of the *Saccharomyces sensu stricto* taxon (Rodríguez-Cousiño et al., 2017), cross-species LA virus transmission between *S. cerevisiae* and *T. delbrueckii* seems improbable. Unfortunately, we have no LA virus sequences from different *Saccharomyces* species isolated from the same location or locations that are very close geographically in order to analyze in depth whether cross-species transmission of LA viruses between yeasts of the *Saccharomyces sensu stricto* group actually occurs.

Our finding that the sequence identity of ScV-LA viruses from *S. kudriavzevii* and *S. uvarum* with some *S. paradoxus* viruses is greater than that between some *S. paradoxus* strains themselves indicates possible cross-species transmission among closely related yeasts (such as those of the *Saccharomyces sensu stricto* taxon) when they coincide in the same habitat, as has previously been suggested (Rodríguez-Cousiño et al., 2017).

As mentioned above, an associated co-evolution of specific LA virus variants with the corresponding specific type of M virus has been suggested based on a possible role of each killer toxin selecting the LA variants that best support each specific toxin-encoding M virus. This is a plausible hypothesis to explain why LAlus4 is specifically associated with Mlus4 virus (Rodríguez-Cousiño et al., 2013) and that LA2 is required for specific M2 maintenance, whereas neither LA nor LAlus4 show helper activity for M2 with the same genetic background (Rodríguez-Cousiño and Esteban, 2017). Our results, however, do not support this hypothesis because the same ScV-LAlus virus found in all strains of the *S. cerevisiae* cluster, whose Gag-Pol amino-acid sequence varied only by 0–1.5%, supports various M virus types (M1 in EX231, M2 in EX1125, and Mlus in EX229, EX436, and EX1160). These results indicate that there was no associated co-evolution of specific LA with specific M viruses at all. It seems that an M virus can infect new yeasts and be stably maintained inside the cell as long as a given LA virus provides it with the required helper activity.

TdV-LAbarr1 is the most distant from the rest of the viruses (62–63% identity), even from those viruses from *S. cerevisiae* strains isolated in the same location. Therefore, the co-evolution can be hypothesized of a specific LA virus with its specific host and habitat. The sequence identity percentage of TdV-LAbarr1 with its *Saccharomyces* counterparts was less when comparing only Gag sequences, but greater when comparing only the RdRp domain sequences. This indicates that LA viruses may have evolved to adapt their capsid’s functioning to better ensure replication in different yeast species and habitats. On the contrary, as features of the RNA polymerase are strongly conserved, no great changes in this enzyme would be required for these viruses to replicate in unrelated yeast species.

### Features of 5′- and 3′-Extra Sequences Found in LA Genomes

The extra sequences we found are probably only part of the actual extra sequences that might be present in each virus, and we cannot be sure whether they are present in the dsRNA within the completed virion or just part of an RNA intermediary of the virus. Similar results have been reported for yeast M viruses sequenced using HTS techniques (Ramírez et al., 2017). In that case, however, no viral self-identity was found between the extra and canonical sequences of the same virus, ribosomal RNA sequences were only found in the 3′-extra sequences, and sequences from other organisms (such as *S. cerevisiae* LBC-2 virus, wine grape, *Saccharomycopsis fibuligera*, and melon) were also found in the 5′-extra sequences. It was suggested in that work that ScV-M RNA could somehow promiscuously covalently join other host viral or cellular RNAs, as has also been suggested for poliovirus RNA (Gmyl et al., 2003) and plant viruses (Sztuba-Soliñska et al., 2011). In this way, M viruses could stay integrated in cellular RNA as rRNA, similarly to the case of retroviruses and retrotransposons in chromosomal DNA, protecting themselves from disappearance under potential stressing conditions as long as the receptor RNA remains in the cell.

Our new results do not contradict that hypothesis, but do suggest new possibilities to explain the existence of extra sequences beyond the canonical ends of LA viruses. The presence of identical stretches in the 5′-extra sequences of some LA genomes (LA2-EX1125 and LAlusA-EX1160, or LA1-EX231 and LAlus1-EX436) suggests that at least part of these extra sequences may have a common origin. Given that viral self-identity is frequently found between some stretches of extra and canonical sequences, the presence of extra sequences may be a collateral result of some imprecise molecular mechanism involved in the viral replication cycle—cap-snatching, for example (Fujimura and Esteban, 2011). This circumstance may favor LA RNA recombination with other RNA and which may fulfill some still unknown structural feature. As mentioned above, this possibility could provide these viruses with a strategy to protect themselves from disappearance under strongly stressing conditions, as long as they stay bound to a less vulnerable host RNA molecule. This phenomenon could be similar to the endogenization of certain ant genome RNA viruses (Flynn and Moreau, 2019), but, in yeasts, employing a different strategy that may involve rRNA instead of nuclear chromosomes. Alternatively, the formation of double-strand stem loops at the ends of these virus genomes may protect the intermediary ssRNA from degradation by single-strand exonucleases, or also provide a free 3′-end in the ssRNA to be used by primer-dependent RNA-dependent RNA polymerases for double-stranded RNA synthesis. These double-strand stem loops might even have both functions at the same time. Moreover, the formation of kissing stem loops may help maintain part of the viral genome temporarily unpaired so as to facilitate the accessibility of polymerase to an (−)ssRNA template for mRNA transcription.

Intramolecular interaction between extra sequences and proximal canonical sequences (such as the possible kissing stem loops found in TdV-LAbarr1 and ScV-LA1-EX231) may play an as yet unknown role in the biology of these viruses (Lim and Brown, 2018). Beyond these intramolecular interactions, the presence of terminal rRNA sequences in 5′- and 3′-extra sequences of yeast viruses could be involved in intermolecular interactions related to some biological process of these viruses. Indeed, rRNA-containing mRNAs have been found extensively in mammal cells. Among these, short rRNA sequences seem to function as *cis*-regulatory elements in translational efficiency, and large portions or even almost entire sequences of rRNA may have functional significance for some neurodegenerative diseases (Mauro and Edelman, 1997; Kong et al., 2008; Pánek et al., 2013). Moreover, as the portions of rRNA found in yeast viruses are different and do not share homology, sequence stretches in the same or different viruses such as LA and M could interact in a similar way to how they interact in the ribosome, maybe even involving ribosomal proteins as has been suggested for rRNA-like sequences and rRNA interaction for *cis*-regulation events (Mauro and Edelman, 1997). This also raises the possibility of a ribonucleoprotein being created that may resemble the yeast ribosome and contain the virus genome. This ribosome-like complex may also be a strategy of these viruses to ensure that they remain in the yeast cell, or it may be related to some other, still unknown, biological function.

## Conclusion

The killer dsRNA virus system of *T. delbrueckii* Kbarr1 yeast seems very similar to that previously described for *S. cerevisiae*. The autonomous LA viruses from the two yeast species show high nucleotide sequence identity, especially in the most relevant functional motifs, which indicates that they are phylogenetically related. LA virus transmission among yeasts of the same species living in the same geographical location seems to be feasible, but not cross-species transmission among phylogenetically distant yeasts such as *T. delbrueckii* and *S. cerevisiae*. Co-evolution of LA and M viruses does not seem likely, although co-evolution of LA virus with a given yeast species may occur in a specific location or habitat. Extra sequences located up- and down-stream from the viral canonical genome may form interesting RNA secondary structures, which could be involved in virus maintenance by avoiding ssRNA degradation and facilitating dsRNA synthesis.

## Data Availability Statement

The datasets generated for this study can be found in the online repositories. The names of the repository/repositories and accession number(s) can be found in the article/Supplementary Material.

## Author Contributions

MR conceived the project, analyzed the data, and wrote and edited the manuscript. MR, RV, AM, and MM designed and performed the experiments. All authors contributed to the article and approved the submitted version.

## Conflict of Interest

The authors declare that the research was conducted in the absence of any commercial or financial relationships that could be construed as a potential conflict of interest.

## Footnotes

1 http://unafold.rna.albany.edu/?q=mfold/RNA-Folding-Form

2 http://rna.tbi.univie.ac.at/forna/
