# Supplementary material for: Genome Organization of a New Double-Stranded RNA LA Helper Virus From Wine Torulaspora delbrueckii Killer Yeast as Compared With Its Saccharomyces Counterparts
Source: Front Microbiol. 2020 Nov 23;11:593846. doi: 10.3389/fmicb.2020.593846 (PMC7721687; doi:10.3389/fmicb.2020.593846)
Supplement: Supplementary file 1 [file Presentation_1.pdf]

Supplementary Material

1.1 Supplementary Figures

```
LA1-ori   --------------GAAAAATTT-----------------TTAAATTCATATAACTCCCC 29
LAlus4    --------------GAAAAATTT-----------------GAATAATCATATAACTCCCC 29
LAbarr1   CACGTAGCTTTATTAATTAATATGCTACGTGCCTATCATCTCAAAGTCCTATTGATCCCC 60
                        .*::***:*                   *:* **.***:..*****
                       5' conserved
LA1-ori   ATGCTAAGATTTGTTACTAAAAACTCTCAAGATAAATCGTCTGATCTATTCTCTATTTGT 89
LAlus4    ATGCTTAGATTCGTTACCAAAAACTCTCAAGACAAGTCCTCCGATTTATTTTCTATTTGC 89
LAbarr1   ATGTTCAGATTTATTAACAGAAAAACTACG---CAAGCTAATAACATCATCCCTATCCAG 117
          *** * ***** .***. *.***.:**...   .*. * :. .*  *.:*  ****  .
          Start of Gag and Gag-Pol
LA1-ori   TCTGATCGCGGAACTTTTGTTGCCCATAATAGAGTTCGGACTGATTTCAAGTTTGACAAC 149
LAlus4    TCTGACAAAGGTACTTTTGTTGCGCATAATAGGGTGAGGACTGACTTTAAATTTGACAAC 149
LAbarr1   TCGGATCAGGGAACTTTTACGATATACGGTAGGAATCGTATTGACTTCAAGTATGATGGC 177
          ** ** .. **:******.  .   * ..***..: .* * *** ** **.*:*** ..*

LA1-ori   TTAGTATTCAACCGAGTTTATGGTGTTTCTCAAAAATTTACGTTAGTCGGTAACCCGACA 209
LAlus4    TTAGTTTTTAATCGAGTCTACGGCGTGTCACAAAAGTTCACCCTAGTCGGCAACCCGAAA 209
LAbarr1   CTAACTTTTTCTAGGCAATTAGGAGTTTCCCAGAAATTTACTTTGCTTGGTAACCCGGAT 237
           **. :** :. .*. : *: ** ** ** **.**.** **  *. * ** ******..:

LA1-ori   GTCTGCTTTAATGAAGGCAGTTCTTACCTAGAAGGTATTGCTAAAAAGTACCTAACTTTG 269
LAlus4    GTCTGTTTCAATGAGGGAAGTTCGTACTTGGAGGGTATTGCAAAGAAATATCTGACCTTG 269
LAbarr1   GTCCACCAAAGTGAGTCGGGCTCCTACTTAGATGGTATTGCGAAGAAGTATTTAACTCTT 297
          *** .  : *.***.   .* ** *** *.** ******** **.**.**  *.**  *

LA1-ori   GATGGAGGACTTGCCATTGACAATGTCCTCAATGAGTTGAGATCCACTTGCGGTATCCCA 329
LAlus4    GACGGTGGCCTGGCCATTGATAACATCCTCAATGAATTGAAATCAACATGTGGTATACCA 329
LAbarr1   GAGGGAGGACTAGCTATTGAGAATGTCATGTCTGAATTACGCAGTAACAGCGGGATACCT 357
          ** **:**.** ** ***** ** .**.* :.***.**....:  *. :* ** **.**:

LA1-ori   GGTAATGCTGTTGCATCTCATGCGTATAATATTACATCCTGGCGTTGGTATGACAATCAC 389
LAlus4    GGTAATGCTGTCGCTTCTCATGCATACAACATTACGTCTTGGAGATGGTACGATAATCAC 389
LAbarr1   GCCTCAGCCGTATCAGCTCACGCGTTTAACATAGCATCTTGGCGCTGGTACGATAACCAC 417
          *  :.:** **  *: **** **.*: ** **:.*.** ***.* ***** ** ** ***

LA1-ori   GTGGCACTGTTGATGAACATGTTGCGTGCTTACCACTTACAAGTATTGACCGAACAGGGC 449
LAlus4    GTGGCATTACTCATGAATATGTTACGTGCGTACCATCTTCAAGTGTTATCTGAACAAGGC 449
LAbarr1   GTAGCTTTATTAATTAATATGCTACGTGCCTATCATCTCAAAGTCCTAGACGAGAGGGGT 477
          **.**: *. * ** ** *** *.***** ** **  * .****   *. . **....**

LA1-ori   CAATATAGCGCTGGAGATATCCCTATGTACCATGATGGACATGTCAAAATCAAGCTACCA 509
LAlus4    CAGTATTCTGCCGGTTCATACCCTATGTACCACGATGGGCATGTCAAAATTAAACTTGAC 509
LAbarr1   GAGTTCGGTGCGGGCCACTACCCTAAGTATGACGATGGGCATCTGGCCATTACTGTAGTG 537
           *.*:    ** **  . ::*****:***  * *****.*** * ...** *.  *:

LA1-ori   GTGACTATCGATGACACGGCAGGCCCAACACAATTCGCTTGGCCTAGTGACAGGTCTACT 569
LAlus4    ACTCCTATCAGTGAGGATGATGCTCCTGACTCATTTAAGTGGCCGAGTGACCGGACAACA 569
LAbarr1   CCACCTACTGGTGCCTTG---GAAACTGACCACTTTAGATGGCCATTCAAAAGAGGTGAA 594
             .***  ..**.       *  .*:... ..** .  ***** :  .*..*.  :..:

LA1-ori   GATTCGTATCCTGATTGGGCACAGTTTTCTGAATCATTTCCATCAATCGACGTCCCGTAC 629
LAlus4    GACACTTACCCAGACTGGGCTCAGTTCTCAGAGTCCTTCCCGTCTATTGACGTCCCATAC 629
LAbarr1   GTGCTCGCACCAGAGTGGGCCCACCATACTGAGGCCATGCCTACAATAGAGGTGCCTTAC 654
          *:     . **:** ***** **  : :*:**. *.:* ** :*:** ** ** ** ***

LA1-ori   CTAGATGTTAGGCCATTGACCGTAACGGAAGTCAATTTCGTGCTTATGATGATGAGTAAG 689
LAlus4    TTAGATGTCAGGCCACTTACTGTTACCGAAGTTAATTTTGTGTTAATGATGATGAGTAAG 689
LAbarr1   ATCGATGTCCGCCCTTTAACATCAGTTGAAGTCAACTTCGTGTTGATGATGATGTGTAAG 714
           *.***** .* **: * **   :.  ***** ** ** *** * *********:*****

LA1-ori   TGGCATAGACGTACTAACTTAGCGATAGACTACGAGGCACCCCAACTAGCTGATAAGTTC 749
LAlus4    TGGCACCGTCGGACTAACCTTGCTATTGACTATGAAGCGCCGGCGCTGGCAGACAAATTC 749
```

```
LAbarr1  TGGTCACGCCAGACTAATCTTGCCATAGACTTCGATTTACCTAACCTGACAGACCGGTTC 774
         *** . .* *. *****  *:** **:****: **    .**   . **..*:** ...***

LA1-ori  GCTTACCGCCATGCGCTTACTGTTCAAGACGCTGACGAGTGGATAGAAGGCGATAGAACT 809
LAlus4   GCCTACCGTCACGCCATTACGGTCCAGGATGCAGATGAATGGATTGAAGGTGATAGGACA 809
LAbarr1  GCCTATAGACACACCGCCAAGGTGACGGAAGCGGACGAGTGGTTAATGAACGAAAGGCCT 834
         ** ** .* ** .*    *. ** ...** ** ** **.***:*:.:... **:**..*:

LA1-ori  GATGACCAGTTCCGCCCCCCCTCGTCTAAAGTAATGTTATCGGCACTTCGTAAGTACGTG 869
LAlus4   GATGATCAGTTTAGACCACCTTCTTCTAAGGTAATGATGTCGGCATTGCGTAAGTATGTT 869
LAbarr1  GATGCAGCCTTTCCGGTACTGTCCAGTAAGGCTATGTTGTCTGCGATCAGGAAGTACGTC 894
         ****.  . ** .     .*  ** : ***.* :***:*.** **. * .* ***** **

LA1-ori  AACCATAACAGGCTGTACAATCAGTTTTACACTGCAGCACAACTGTTAGCTCAAATTATG 929
LAlus4   AATCATAATCGACTTTACAACCAGTTCTATACGGCCGCTCAACTATTAGCTCAGATTATG 929
LAbarr1  AATCACAACAGAGTGTACAATCACTTTGAAACTGCGGCGCAGATACTAGCACAGCTGATG 954
         ** ** ** .*. * ***** ** **  * ** ** ** **..*. ****:**..* ***

LA1-ori  ATGAAACCTGTCCCTAACTGCGCTGAGGGCTACGCTTGGCTGATGCATGACGCATTGGTC 989
LAlus4   ATGAAACCAGTACCTAACTGCGCTGAAGGGTATGCATGGTTGATGCACGATGCTCTGGTC 989
LAbarr1  GTTAAGCCTTTGCCGAATAACGCTGAAGGGCACGCATGGTTACTACATGACCCGGTTGTG 1014
         .* **.**: * ** ** :.******.**  * **:*** *..*.** **  *  * **

LA1-ori  AATATACCAAAATTTGGGTCTATTCGAGGAAGGTACCCCTTTTTGTTATCAGGTGATGCA 1049
LAlus4   AACTTGCCGAAGTTCGGGTCCGTTAGAGGGCGTTACCCGTTCTTACTTGCAGGTGACGCT 1049
LAbarr1  AATATACCTAAGTTCGGGTCAGTTAGGGGCAGATATCCGTTTTTACTCACGGGTGAAGCT 1074
         ** :*.** **.** ***** .**.*.** .* ** ** ** **. *  *.***** **:

LA1-ori  GCGTTGATTCAGGCTACAGCCCTAGAAGACTGGTCTGCTATCATGGCGAAACCCGAGCTG 1109
LAlus4   GCCCTGATTCAGGCAACAGCACTTGAAGACTGGTCAGCAATTATGGCTAAGCCAGAACTG 1109
LAbarr1  TCTTTGGTGCAAGCAACAGCACTAGAAGATTGGTCAGCTATCATGGGTAAGCCGGAGATA 1134
          *  **.* **.**:*****.**:***** *****:**:** ****  **.** **..*.

LA1-ori  GTGTTCACTTACGCGATGCAGGTGTCAGTAGCGTTAAACACCGGACTATACTTACGTCGC 1169
LAlus4   ATTTTTACCTACGCTATGCAGGTTGCTGTCGCGTTGAACACTGGCTTATACTTACGGAGG 1169
LAbarr1  GTTTTTACCTATGCAGTAGATCATGCTATAGCAGTGAATACTGGCTTGTACTTGAGGCGC 1194
         .* ** ** ** ** .*. *   :  *:.*.**. *.** ** **. *.*****..* .*

LA1-ori  GTTAAGAAAACAGGCTTCGGCACAACTATAGATGACAGCTATGAAGATGGAGCGTTTTTG 1229
LAlus4   GTCAAGAAAACTGGTTTCGGTACCACTGTTGACGATAGTTATGAAGACGGCGCATTTCTA 1229
LAbarr1  ATCAAGAAAACGGGAATGGGCACCAGGGTTGATGATAGCTACGAAGATGGTGTCTTCCTT 1254
         .* ******** ** :* ** **.*   .*:** ** ** ** ***** ** *  **  *

LA1-ori  CAACCGGAGACGTTCGTTCAGGCCGCACTAGCATGTTGTACCGGACAAGATGCGCCCCTA 1289
LAlus4   CAACCTGAGACCTTCGTACAAGCTGCGATAGCTTGCTGCACTGGCCAAGATGCACCGCTT 1289
LAbarr1  TCACCTGAGACGTTCATGGCTGCAGCAGTCGCTTGTGCCACCGGAGAGGATGCACCTCTA 1314
          .*** ***** ***.*   . ** **. *.**:**     ** **. *.*****.** **:

LA1-ori  AATGGGATGTCAGATGTGTATGTCACTTATCCAGATCTTCTAGAATTTGATGCTGTTACA 1349
LAlus4   AACGGTATGTCTGATGTGTACGTGACATACCCAGATCTCCTTGAGTTCGATACTATAACA 1349
LAbarr1  AACGGAATGTCAGACGTCTACGTATTTTATCCTCAATTTGTGGATCTCGACACTGTGACT 1374
         ** ** *****:** ** ** ** : :** **: *: *  * **  * ** .**.* **:

LA1-ori  CAAGTACCCATCACGGTCATTGAGCCCGCTGGCTATAACATTGTTGATGATCATTTAGTG 1409
LAlus4   CGTGTCCCTGTTACGGTACTAGAACCTGAAGGCTATAATATACAGGATGGCGCCTTGGAA 1409
LAbarr1  TATATACCTGCCCAAGTTCGTGAAGCGGAAGGGTACAACGTCACTAGCGAGGGTCTTAAA 1434
          .:.*.** .  ...** . :**. * *.:** ** ** .*    .. *.     * .:.

LA1-ori  GTTGTGGGTGTACCTGTGGCATGTTCACCATACATGATATTTCCAGTAGCTGCGTTTGAT 1469
LAlus4   GTGACAGGTGTTCCGATTGCCTGCTCGCCTTACATGATTTTCCCTGTCGCAGCTTTCGAT 1469
LAbarr1  GTTTTGGGAGTACCTTTGGCTTGCTCGCCAGTGTTACTCTACCCTATGGCGGCATTTGAT 1494
         **    .**:**:**  * ** ** **.**: : :*..* *: **:.* ** ** ** ***

LA1-ori  ACTGCAAATCCTTACTGTGGGAATTTTGTCATTAAGGCTGCTAACAAGTATCTCCGTAAG 1529
LAlus4   GAAGCTAACCCCTATTCTGGTAGTTTTGTTATTAAGCCTGCGCTGAAATACCTACGCAAA 1529
LAbarr1  GATGCAAACCCATATTCGGGATCGTTCAAGATCGGCAAAGCCGAAAGGTATGTGAGAGGC 1554
         ..:**:** ** ** *  ** :   ** .: **  ..   .:**  : *..**  * .* ..

LA1-ori  GGTGCCGTGTATGATAAACTCGAAGCATGGAAGTTGGCCTGGGCACTGAGGGTAGCCGGG 1589
LAlus4   GGTGCTCTGTATGCCAAACTAGAGGCCTGGAAACTGGCCTGGGCTATGAGGATTGCAGGA 1589
LAbarr1  AGGGCCGTTTATTCACCATTCGAGGCCTGGAAGCTAGCCTGGGCGGCGAGAATCGCTGGG 1614
         .* **  * *** . ..* *.**.**.*****. *.********   ***..* ** **.
```

```
LA1-ori   TATGACACTCACTTCAAAGTGTATGGCGATACACACGGCTTAACTAAGTTCTATGCTGAC 1649
LAlus4    TACGACACTGAATTTAAAGCTTGTGGCAACACGCATGGTTTATCCAAATTTTATGCTGAT 1649
LAbarr1   TATGATACTCGCGTATCTATACCTGGCGACAATACCGGCTTATCTAAGCTATATGCAGAC 1674
          ** ** *** .. * :.:.    ****.* *. .. ** ***:* **. * *****:**

LA1-ori   AACGGTGACACATGGACACACATACCTGAATTTGTCACTGACGGTGACGTGATGGAAGTA 1709
LAlus4    AATAGCGATAGTTGGACACACATACCGGAATTCGTTAGTGATGAAGAAGTTACGGAAGTG 1709
LAbarr1   AATGCCGATTCCTGGACTCACATACCGGACTCATTTCTCAATCCTGAAGCAGAATTTATT 1734
          ** .  ** :  *****:******** **.*   * .  .*   :**.*  . . ::.*

LA1-ori   TTCGTTACTGCCATCGAACGCAGAGCTAGACATTTCGTTGAACTACCTAGACTGAATTCA 1769
LAlus4    TATGTAACTAATATTGAACGTAGAGCCAGACACTTTGTTGAGCTACCAAGACTAAATTCT 1769
LAbarr1   TATGTGACTGATCTAGAGAGGAGGAGAAGGCACTTTATAGACTTGCCGAACATGACCAAT 1794
          *: ** ***.. .* **..* **..  **.** ** .*:**  *.** *...*.*. :.:

LA1-ori   CCAGCATTCTTCAGATCTGTAGAAGTCAGCACCACTATATATGATACTCATGTGCAGGCT 1829
LAlus4    CCAGCCTTTTTTAAACCAGTAGAGGTAAGCACCGCAATTTACGATACTTACGTACAGGCA 1829
LAbarr1   CCTTCATTCGCAAGATCGATAGAAGTACAGATTACACTTAACGATATGTATGTCGAGTCT 1854
          **: *.**    *.* * .****.**... *  .*:.*::* ****   * **  ** *:

LA1-ori   GGTGCGCATGCGGTGTATCATGCTAGTCGAATCAATCTTGATTATGTTAAGCCTGTTTCG 1889
LAlus4    GGCACATTCTCTGTCTACCATGCAAGCAGGATTAATTTGGATTACGTCAAGCCTGTATCT 1889
LAbarr1   ACTCCCGGGTCCCGCTCACGGGCTATGGTGCTGAGCAGGGACTACGTCGCGCCGATAGCG 1914
          .   *     *    *. *. **:*    ..* *.    ** ** ** ..*** .*: *

LA1-ori   ACCGGCATTCAGGTGATCAATGCGGGCGAACTTAAGAACTACTGGGGTAGTGTGCGTCGT 1949
LAlus4    GCAGGCATACAAGTCATTAACGCTGGTGAACTAAGGAATTACTGGGGTAGTGTACGCCGT 1949
LAbarr1   TCTGGTATACAAGTGATTAGTGCCGCTGACTTGAGGAACTATTGGGGCTCAGTCCGCCGG 1974
           * ** **:**.** ** *. ** *  **. * *.*** ** ***** : :** ** **

LA1-ori   ACTCAGCAGGGTTTAGGAGTGGTAGGTCTTACGATGCCAGCTGTAATGCCTACCGGAGAA 2009
LAlus4    ACGCAGCAGGGTTTAGGAGTGGTAGGTCTTACGATGCCAGCCGTAATGCCTACCGGAGAA 2009
LAbarr1   TCGAATGCGGGTTTATCCTTGGTAGGTTTTACGATGCCAGCAGTAATTCCTACCGAAAGG 2034
          :* .*  .*******  . ******** ************ ***** *******.*...
```

-1 Frame-shift site *Stem loop for frame-shift (ΔG=-14.0 kcal/mol)*

```
LA1-ori   CCTACAGCTGGCGCTGCCCACGAAGAGTTGATAGAACAGGCGGACAATGTTTTAGTAGAG 2069
LAlus4    CGTACAGCTGGCACTGCCCACGAAGACTTGATCGAACAGACGGAAAATGTTTCAGTCGAG 2069
LAbarr1   AGTATCGCTGGTCAAGACCTCCAAGAGGCGATAGAACTAGAGCAAGAGCCTTCAGTAGAA 2094
          . ** .*****  .:*.**:* ****   ***.****:...* *..*   ** ***.**.

LA1-ori   TAAACGTAATCGAACCCTCACACGGACCCCGCCCTACAAGGTACATACTGCAGGAACCAG 2129
LAlus4    TAAACATAATAGAACCATCCCATGGGCCTCGTCCGATGAGATACATATTGAACGAGCCAA 2129
LAbarr1   TAAATCTTATAGAAAGCGAGCACGGTCATAGGCCACGCAGATACATTGCTTCTGGTAATG 2154
          ****  *:**.***. . . ** ** *. .* **  .   **.*****:    . *. ..:.
```

Stop of Gag

```
LA1-ori   GTACGTACCCTGCGTGGATTAGATTCAGGAACAGAGTACAAGCTGTATCGCGTCAGAAAG 2189
LAlus4    ATACATACCCCGCATGGATAAGGTTCAGAAATAGAGTACAGGCGGTATCTAGACAGAGGG 2189
LAbarr1   AAGTAGTCCCTATATGGATCAGATTCGGCCGCCGCATTGAGTGTGTTTCACGTCAGAAAG 2214
          .:. . :*** . .***** **.***.* .. .*..*: *.   **:** .*:****..*

LA1-ori   CCACTCACTTCTTGTTTGACATCGTACCTGCCGCAGTAATTAGTGATTTTACTACGTCTG 2249
LAlus4    CAACACATTTCTTATTTGATATAGTACCGGCCAGCAAAATTGCGGACTATACTACTTCAC 2249
LAbarr1   CAACTTACTTTCTGGCTGACGTTATCCCCAGAGAAGAGGCTATAAACTACACCTCTGCTA 2274
          *.**: * **  *.  *** .* .*.** . .. ..:.. *.  .* *: ** :*  *:

LA1-ori   ACACGTCTTCGTTTGCATACAAATCGCACACCTACGCTGTAAATGTAACAGCATTGAGGT 2309
LAlus4    AAACGGCTACTTTTGCATACAGGTCGCACACGTATGCATGCACGGTAACGGCACTACGCT 2309
LAbarr1   ACACAGCCCAAGTGGCCATAAGGAGAAGTACATATACGGCTAGCGGTATTTTAGTCAAAC 2334
          *.**. *  .  * **.::.*..: ... ** ** .*    *  * :*    * * ..

LA1-ori   TCAGTGACACTTATGCCTTGTACGTACAGACTGATACCAACATGACAATTTTAAGCCCAG 2369
LAlus4    TTGGAGATGACTACGGCTTGTATGTCCAGGTAGAGGCTAACATGACATTGTTAAGTCCGG 2369
LAbarr1   TCGGAGACAACTATGGTTTCTACCTACACGTCGATGAAGATATGACAGTCAAAACGCCGG 2394
          * .*:** .. ** *  ** **  *.** .  ** .. .* ****** * ::**  **.*

LA1-ori   CGGCGCGTCGCCAGGCTTCTGCGACGTACTCACAGGTGGCAGGGTTTTGTTATAACACAC 2429
LAlus4    CTGCACGGCGACAAGCTTCTGCTACGTACTCTCAGGTTGAGGGGTTCTGTTTTAACACAC 2429
LAbarr1   CCTTACGAAGGTACGCTTCGATGGCTTACTCTAACGTGCGCGGTTCATGTTATAACACTC 2454
          *   .** .*  * *****  .  .* *****:.* **    ** *  ****:******:*
```

```
LA1-ori  CTACCGTTATGGATTCGCTAGCGAATATCTTGGACGTAGACCGCAATATACGACCCAAAC 2489
LAlus4   CTACGGTCATGGACACGCTAGCTAACATCTTGGATGTTGACCGCAATATTAGACCAAAGC 2489
LAbarr1  CTACCATAATGGACACTCTGTGTAATATTTTTGACTACGACCCCGATTCGCGGCCATCCT 2514
         **** .* ***** :* **.   ** ** ** **  : **** *.**:  .*.**.:.

LA1-ori  ACTTCAAGGGTTTACGGCTATACACAAGGTCTAAGGTCACTGCTCAACATCATACTCACT 2549
LAlus4   ACTTTAAAGGTCTGCGTACTTATGAGAGGTCTAAGGTCACAGCACAACACCACACTCACT 2549
LAbarr1  CATTTAAGGGACTGCGTTCTTACACCAAGTCAAAGGTTACGGGGATGCACCATACCCATT 2574
         ..** **.**: *.**   :** .. *.***:***** ** *  .:.** ** ** ** *

LA1-ori  TGCGGCCAGACGAGCTAGTGGAAGCGGCCGCAAAGGTCTCGCCTAGACGTAAATACTACT 2609
LAlus4   TGCGGCCTGATGAAGTACTGGAAGCAGCGGCACGTGTATCGCCTAGGCGAAAGTACTACT 2609
LAbarr1  TAAGGCCACAAGATGTTTTGTCGGCTGCCAAGAAGACCGGAAATAGTCATTATTACTACG 2634
         *..****: * **  *: ** ..** ** ..... . .  ...*** *.::* ******

LA1-ori  TAATGTGTGTAGTTGAGCTGCTCGCGAACTTACAAGTAGATCTTGAAGCAGCAGTAGCTA 2669
LAlus4   TATTATGCGTCGTAGAGCTATTAGCGGCATGCGAGGTTAACATTGAAGCAGCAGTAGCTA 2669
LAbarr1  TGCAGGTGGCTGTCGAGCGTCTAGCATCGGCTGGCGTAACTACAGAGGCAACTGTTTCAA 2694
         *. :.   *  ** ****   *.**. .     . **:.. . :**.***.*:**: *:*

LA1-ori  CTATTCTCGCATATGTCCTGACACTAAGTGAAAAATTTGTACCAATTTTCTTGGATTCTA 2729
LAlus4   CAATAATGACGTACGTACTCACTTTGGACGAAAAGTTCATACCTTTGTTTCTAGACTCAC 2729
LAbarr1  CCATAATGTTGTATGTCCTCACATCAAACGACACCACACGGGCATTGTTCCTAGATTCTA 2754
         * **:.*   .** **.** **:  ... **.*. :    . *::* **  *.** **:.

LA1-ori  GAGCAATATGGGTCGGTGAGCCTGGGCCTGATGCTCTGACTGCACGTCTCAAGGCCAGTA 2789
LAlus4   GGACAATATGGCAGGGTAGTAAAGGACCTGAAGAACTAACAGCCAGACTCAAGAAGGCGA 2789
LAbarr1  AAGCTATATGGTCGAAGACTGACGGGGAAGATGAATTATCTGACCGACTCAAGCGAGTAA 2814
         ...*:******   .. .   . **. .:**:*.: *.:*:*...*:******   .  *

LA1-ori  GTGGGCAGATCAAGAGCATACACACGGCTGATTACGAACCACTCACTGAACTATTCGAGT 2849
LAlus4   GCGGGCAGATTAAAAGTGTTCATACAGCTGACTATGAGCCACTGACGGAACTGTTTGAAT 2849
LAbarr1  GCGGACAGATCAAGTCAATTCACACAAGTGAGCTGGAGCCTTTAACCGAGTTGTTCGAGC 2874
         * **.***** **.:  .*:** **.. ***  : **.**: * ** **. *.** **.

LA1-ori  TAGCAGTATTGATGAACCGAGGTGTTGGCCATGTCTCTTGGCAAGCTGAAAAGGATCATC 2909
LAlus4   TGGCTGTGCTCATGAACAGAGGCGTCGGGCATGTATCCTGGAAGACTGAAAGAGAACACA 2909
LAbarr1  TTCAGGTCTTGATGAACCGGGGTATAGGACATGTTGACTGGAATGGGGAAAGACATAACA 2934
         *  . **  * ******.*.** .* ** *****   . ***.* .  ****.. *:.* .

LA1-ori  GCTTGAATCCCGACGTGGCTGTAGTTGATCAAGCACGGCTATATTCGTGTGTGCGCGACA 2969
LAlus4   GAGAGAATCCGGATGTAGCAAACGTTAATCAACAAGCACTCTACGCGTGCGTACGTGACA 2969
LAbarr1  GGACCAACCCTAATACTGTGAATGTAGATCAAAAGATGCTCTATAGTGTTGTACGCGATA 2994
         *    ** ** .* .  *  .: **:.***** ..  .**.**       **.** ** *

LA1-ori  TGTTCGAAGGATCAAAGCAGACGTATAAATATCCCTTTATGACGTGGGATGACTACACTG 3029
LAlus4   TGTTCGAGGGGGCCAAGCAGACTTATGATTATCCTTACATGACATGGGATGACTATACGT 3029
LAbarr1  TGTTTGAAGATTCTAAGCAATCGTACAAATACCCGTATATGACATGGAACGACTATGCGG 3054
                 **** **.*.  * *****.:* ** .*:** ** *: *****.***.* ***** .*

LA1-ori  CAAACAGATGGGAGTGGGTTCCAGGTGGCAGTGTCCACTCTCAATACGAAGAAGACAACG 3089
LAlus4   CAAGTCGGTGGGAATGGGTGCCTGGAGGAAGTGTTCATTCTCAGTACTCCGAGGATGATG 3089
LAbarr1  ATAGTAGGTGGGAATGGATACCTGGAGGTAGTGTACACTCGCAATACCCTCAGGATGATG 3114
         .:*. .*.*****.***.* **:**:** ***** ** ** **.***  .  *.** .* *

LA1-ori  ATTATATCTATCCTGGTCAGTATACTAGGAACAAGTTCATAACTGTTAACAAAATGCCCA 3149
LAlus4   AATATATATTTCCAGGACAATATACTAGGAACAAGTTTATTACAGTCAACAAAATGCCTA 3149
LAbarr1  AATACATAGTCCCTGGTCTCTACACCAGGAATAAGTTCATAACAGTAAACCTCATGCCGA 3174
         *:** **. : **:**:*: ** ** ***** ***** **:**:** ***.:.***** *

LA1-ori  AACACAAAATATCTAGAATGATAGCATCACCGCCTGAGGTACGAGCTTGGACGTCGACGA 3209
LAlus4   AGCATAAGATCGCGCGTATGATAGCATCTACGCCTGAAGTTAGGGCATGGACCTCAACGA 3209
LAbarr1  AGAATAAGTTAGCCTCAATCGGCGCATCGAAACCAGAAGTCAGAGCATGGACATCAACTA 3234
         *..* **.:*. *   :** . .***** ...**:**.** .*.**:***** **.** *

LA1-ori  AGTACGAATGGGGCAAGCAACGTGCTATCTACGGGACGGATCTACGAAGTACACTGATAA 3269
LAlus4   AGTATGAATGGGGTAAACAAAGAGCTATATATGGCACAGACCTACGGAGCACACTTATCA 3269
LAbarr1  AATACGAGTGGGGGAAACAGAGAGCTATCTATGGAACTGATCTGAGGAGCACTCTGATAA 3294
         *.** **.***** **.**..*:*****.** ** ** ** **..*.** **:** **.*

LA1-ori  CTAACTTTGCAATGTTCAGGTGCGAGGATGTTCTCACTCACAAGTTCCCAGTAGGCGACC 3329
LAlus4   CTAACTTCGCTATGTTCAGATGTGAAGATGTGTTAACACATAAATTTCCTGTAGGTGATC 3329
```

```
LAbarr1  CTAATTTTGCGATGTTCAGATGCGAGGACGTACTTAAACACAAGTTTCCTGTTGGCGATC 3354
         **** ** ** ********.** **.** **  * *.:** **.** **:**:** ** *

LA1-ori  AGGCAGAGGCAGCAAAGGTGCACAAACGGGTGAACATGATGCTGGACGGTGCCTCTAGTT 3389
LAlus4   AAGCTGAAGCTGCGAAGGTCCACAAACGTGTCAACATGATGCTAGACGGTGCATCGAGTT 3389
LAbarr1  AAGCAGAAGCAAGTAAGGTTCATAAAAGGATTTCCATGATGCTAGGGAATGCGTCTAGTT 3414
         *.**:**.**:.  ***** ** ***.* .* :.*********.*. ..*** ** ****

LA1-ori  TCTGCTTCGATTATGATGACTTCAATTCTCAGCATTCAATAGCTAGTATGTATACGGTTT 3449
LAlus4   TCTGTTTTGATTACGACGACTTTAATTCCCAGCATTCAATAAGCAGCATGTACACAGTAC 3449
LAbarr1  TTTGTTTCGATTATGACGACTTCAATTCTCAGCACTCTATTTCGAGCATGTACACTGTGT 3474
         * ** ** ***** ** ***** ***** ***** **:**:    ** ***** ** **

LA1-ori  TGTGCGCTTTCAGGGACACATTTAGTCGCAACATGTCTGATGAACAAGCAGAGGCGATGA 3509
LAlus4   TATGTGCTTTCAGAGACGCTTTCACACGTAATATGTCCATTGAACAACGCGAGGCTATGG 3509
LAbarr1  TGTTGGCGTTCAGGGATGCTTACTCTCGTAATATGTCGCCAGCTCAGTTGCGTGCGATGG 3534
         *.*  ** *****.** .*:*: : :** ** *****   :*.:**.     . ** ***.

LA1-ori  ACTGGGTGTGTGAGTCCGTCAGACACATGTGGGTACTAGATCCTGATACCAAGGAGTGGT 3569
LAlus4   ATTGGGTATGTGAGTCAGTCAAGCACATGTGGGTTTTAGATCCGGATACTAAGACTTGGT 3569
LAbarr1  ATTGGGTATGTGAATCAACCAAACATATGTATGCTAAGGACCCGGGGACAAACGACTGGT 3594
         * *****.*****.**..  **..** ****. * : :.** ** *. ** **  .. ****

LA1-ori  ACAGACTACAAGGTACATTACTGTCAGGATGGCGGTTAACCACATTTATGAACACTGTGC 3629
LAlus4   ACCAATTGAAGGGCACCTTGTTATCAGGATGGCGACTCACAACATTTATGAACACAGTGT 3629
LAbarr1  ATGAATTGAAAGGAACTTTGTTATCAGGATGGAGACTAACTACGTTTATGAATACTGTTC 3654
         *  .* *..*.** ** **. *.*********.*. *.** **.******** **:**

LA1-ori  TAAACTGGGCGTATATGAAATTAGCTGGCGTATTTGATCTGGATGACGTTCAAGACTCGG 3689
LAlus4   TAAATTGGGCATACAAGAAGCTGGCTGGGGTATTTGATTTAGATGACGTCCAAGATTCAG 3689
LAbarr1  TCAACTGGGCATATATGAAGGTCGCAGGAGTCTTTGACATAACTGACGTAGAAGATTCGG 3714
         *.** *****.** *:***. * **:** **.*****  *...******  **** **.*

LA1-ori  TACACAACGGTGATGATGTTATGATTAGTCTCAACCGCGTGAGCACAGCAGTAAGAATAA 3749
LAlus4   TACATAACGGCGATGATGTCATGATTAGTTTAAACAGAGTCAGTACAGCCGTTAGAATAA 3749
LAbarr1  TGCATAACGGGGACGACGTCATGATAAGTCTAAACCGGGTGTCCACAGCAGTTAAGATAA 3774
         *.** ***** ** ** ** *****:*** *.***.* ** :  *****.**:*..****

LA1-ori  TGGACGCTATGCACCGGATAAATGCGCGAGCACAGCCGGCGAAGTGTAACTTGTTTTCGA 3809
LAlus4   TGGACGCGATGCATAGAATCAATGCTAGGGCCCAGCCGGCTAAGTGTAACCTGTTCTCTA 3809
LAbarr1  TGGAGAGGATGAGGCTGATTAACGCTAGAGCTCAAGAAGCTAAGTGTAATCTCTTTTCAA 3834
         ****  .  ***.. . .** ** **  .*.** **. ..** ********  * ** ** *

LA1-ori  TAAGTGAATTTCTGAGGGTAGAACACGGTATGAGCGGAGGCGATGGTCTTGGGGCTCAGT 3869
LAlus4   TAAGCGAATTCTTACGCGTAGAGCATGGGATGAGCGGAGGTGACGGCTTAGGAGCTCAGT 3869
LAbarr1  TCAGTGAATTCTTACGGGTCGAACATGGTATGACAGGAAAATCTGGTTTGGGTGCACAGT 3894
         *.** *****  *..* **.**.** ** **** .***..  . **  * ** **:****

LA1-ori  ACTTAAGTAGGTCTTGTGCTACTCTTGTACACAGTAGGATTGAGTCTAACGAACCACTGT 3929
LAlus4   ACTTAAGTCGGTCATGCGCCACACTGGTTCACAGCCGTATAGAGTCGAACGAGCCACTAT 3929
LAbarr1  ACCTAAGCCGTTCCTGTGCTACTATAGTGCATAGTAGGATTGAATCAAATGAGCCGATAT 3954
         ** **** .* ** ** ** **:.* ** ** ** .* **:**.** ** **.**..*.*

LA1-ori  CAGTAGTACGAGTTATGGAAGCAGACCAGGCTAGATTGCGCGACCTGGCAAACAGAACGC 3989
LAlus4   CTGTTGTCCGGGTTATGGAAGCAGACAAAACGCGGCTACGGGACTTGGCTAACAGGACCA 3989
LAbarr1  CACTTGTCAGATTACTTGAAGCTGATAAGACCAGGCTGAGGGACCTAAGTGATAGAACGA 4014
         *: *:**..*. *:.* *****:** .*..* .*. *..* *** *.. :.* **.** .

LA1-ori  GGGTACAATCTGCGGTAACAGCGATAAAAGAACAACTCGACAAACGTGTCACTAAGATAT 4049
LAlus4   ACATTAAAGCATCAGTAACAGAAATAGAAGAGCAACTTGATCGTAGAGTCACTTCGATAT 4049
LAbarr1  CACGAGAAGACGTCTTGGTGAACATAAAGTATGAGCTAGATATGAGAGCTGTCAACGTAT 4074
             : ** .     *.. ... ***.*. *  *.** **  .  .*:*  .   :. .***

LA1-ori  TCGGAGTTGGTGATGACGTTGTGCGCGACATACACACAGCTCACAGGGTGTGTGGCGGTA 4109
LAlus4   TCAAAGTGGATAGAGAAGTAGTCAAAGCGATTTCTACAGCTCACAGAGTCTGCGGCGGTA 4109
LAbarr1  TTGGCGCCGATTACAACGTAGTTCAAGACATATATAAATCTCACAGAGTATGCGGTGGGA 4134
         * ...*  *.* . .*.**:** ...*. **: . *.* *******.** ** ** ** *

LA1-ori  TCTCGACTGATACCTGGGCACCGGTTGAAACTAAGATAATAACAGACAATGAAGCATATG 4169
LAlus4   TATCAACCGACCCGTGGGCACCAGTTACTACAAAAATAAAGACAGACAACGAAGCATACG 4169
LAbarr1  TTAACGATGATAAGTGGGGATCAGTAGACACTGAGATTCACACAGATTCTGGGGCATATC 4194
         * :. .. ** .. **** * *.**:.. **:.*.**:.: ***** :. *..*****
```

```
LA1-ori  AAATACCATACGAAATAGATGATCCATCATTTTGGCCAGGGGTAAATGATTATGCTTATA 4229
LAlus4   AAATACCATACGAAATAGATGATCCATCATTTTGGCCAGGGGTAAACGATTATGCTTATA 4229
LAbarr1  AGATACCTGAAGAAATAGACGATCCGTCATTTTGGCCAGGTGTTAACGATTATGCCAGGA 4254
         *.*****: *.******** *****.************** **:** ******** :. *
                      Stem loop for packaging (ΔG=-4.3 kcal/mol)

LA1-ori  AAGTCTGGAAAAATTTCGGAGAACGACTCGAATTTAATAAGATTAAAGATGCCGTAGCTA 4289
LAlus4   AAGTCTGGCAGAATTTCGGCGAAAGGCTGGAGTTTAACAAGATTAAAGACGCTGTTTCAA 4289
LAbarr1  AAGCATACAATATCCTAGGAGAGAAACTAGAATTTAACAAGATTAAGAGTGCAGTAGCCA 4314
         *** .*. .* *:  *.**.**....** **.***** ********... ** **: * *

LA1-ori  GAGGGAGTAGGAGCACTATAGCTCTGAAACGTAAGGCTAGGATAACATCTAAGAAGAATG 4349
LAlus4   AGGGCAGCAGAAACACGATAGCCTTGAAACGTAAGGCGAAGATTTCTGCAGTCAAGAACG 4349
LAbarr1  GAGGTAGCAGGCTAACGATTGCCATGAAACGAAGAGCGCGAGTAGGATCGACGCCTACAG 4374
         ..** ** **.. .** **:**  *******:*..** ....*:  : * .  .. *. *

LA1-ori  AATTCGCTAACAAGTCGGAATGGGAAAGGACAATGTACAAAGCCTATAAGGGTTTGGCAG 4409
LAlus4   ACTTCGTCAATAAATCTGAATGGGAGAGAACTATGTACAAAGCTTACAAAGGTTTAGCAG 4409
LAbarr1  AGTTTATTAATATCAAGCAATGGGAGCGTGCGATGTACAGAGCTTTTAAAGGGGTTGCGA 4434
         * ** .  ** *: :.  *******..* .* *******.*** *: **.**  * **..

LA1-ori  TCTCATACTATGCTAACCTGAGCAAATTCATGAGTATACCACCAATGGCGAACATTGAAT 4469
LAlus4   TATCTTATTATGCTAACTTGAGCAAATTCATGAGCATACCACCCATGGCAAACATAGAAT 4469
LAbarr1  TATCATATTACACCAGCTTGAGCAAATTTATGGCTGTACCACCTATATCAGGGCTTGATC 4494
         *.**:** ** .* *.* ********** ***.  .******* **. *... .*:**:

LA1-ori  TTGGGCAGGCTAGATATGCTATGCAAGCAGCCCTTGATAGTTCTGATCCACTCCGGGCAT 4529
LAlus4   TCGGACAAGCTAGATTTGCGATGCAGGCGGCCTTAGATAGTTCCGATCCTTTAAGAGCAC 4529
LAbarr1  GAGGCGAGGGCGCCATGGCAGTTAACGCTGCTATTGCGAGCGCCGATCCTCTGCGGGCAC 4554
           **  *.*  . .:: ** .* .* ** **  *:*. **  * *****: * .*.***

LA1-ori  TACAGGTCATACTGT-AATTGCCAAAAAGATAATGGGAATTACCCATATGC--------- 4579
LAlus4   TACAAATATTCTTATGAAGTGCTCGAACGATGAGGGTTTTTACCCATATGC--------- 4580
LAbarr1  TTCAAATTCTAGTATAATCACTAGGGAAAGAGATGGATTTAATCCATAAGCATACCACCT 4614
         *:**..*  *. *.* *: :    ..*...:.* ** ::*:* *****:**
                         Stop of Gag-Pol    Stem loop for replication (ΔG=-3.3 kcal/mol)
LA1-ori  --------
LAlus4   --------
LAbarr1  ATATCAGG 4622
```

Figure S1. Multiple sequence alignment between ScV-LA1-original, ScV-LAlus4, and TdV-LAbarr1 (+) strand nucleotide sequences (cDNA). 5´GAAAAA conserved motif (5´ conserved), translation initiation (start of Gag and Gag-Pol, or internal ATG in Pol ORF of LA, LAlus4 and LAbarr1) and termination (stop of Gag or stop of Gag-Pol) codons, ribosome frameshifting site (-1 frameshift site), frameshifting associated sequence (stem loop for frameshift), packaging signal (stem loop for packaging), and replication signal (stem loop for replication) are indicated and shaded in the nucleotide sequence. An AU-rich 15-nucleotide stretch located in the 5´ untranslated terminal region is underlined. ∩, ribosomal frameshift. Asterisks (*), colons (:), and dots (.) indicate identical nucleotide positions, transitions, and transversions, respectively.

```
LA1-ori MLRFVTKNSQDKSSDLFSICSDRGTFVAHNRVRTDFKFDNLVFNRVYGVSQKFTLVGNPT 60
LAlus4  MLRFVTKNSQDKSSDLFSICSDKGTFVAHNRVRTDFKFDNLVFNRVYGVSQKFTLVGNPK 60
LAbarr1 MFRFINRKTT-QANNIIPIQSDQGTFTIYGRNRIDFKYDGLTFSRQLGVSQKFTLLGNPD 59
        *:**:.:::  ::.:::.* **:***. :.* * ***:*.*.*.*  ********:***

LA1-ori VCFNEGSSYLEGIAKKYLTLDGGLAIDNVLNELRSTCGIPGNAVASHAYNITSWRWYDNH 120
LAlus4  VCFNEGSSYLEGIAKKYLTLDGGLAIDNILNELKSTCGIPGNAVASHAYNITSWRWYDNH 120
LAbarr1 VHQSESGSYLDGIAKKYLTLEGGLAIENVMSELRSNSGIPASAVSAHAFNIASWRWYDNH 119
        *  .*..***:*********:*****:*::.**:*..***..**::**:**:********
                                         H154 for 5'cap-snatching + variable region
LA1-ori VALLMNMLRAYHLQVLTEQGQYSAGDIPMYHDGHVKIKLPVTIDDTAGPTQFAWPSDRST 180
LAlus4  VALLMNMLRAYHLQVLSEQGQYSAGSYPMYHDGHVKIKLDTPISEDDAPDSFKWPSDRTT 180
LAbarr1 VALLINMLRAYHLKVLDERGEFGAGHYPKYDDGHLAITVVPPTG-ALETDHFRWPFKRGE 178
        ****:********:** *:*::.**  * *.***: *.:  . .     .  * ** .*

LA1-ori DSYPDWAQFSESFPSIDVPYLDVRPLTVTEVNFVLMMMSKWHRRTNLAIDYEAPQLADKF 240
LAlus4  DTYPDWAQFSESFPSIDVPYLDVRPLTVTEVNFVLMMMSKWHRRTNLAIDYEAPALADKF 240
LAbarr1 VLAPEWAHHTEAMPTIEVPYIDVRPLTSVEVNFVLMMMCKWSRQTNLAIDFDLPNLTDRF 238
           *:**:.:*::*:*:***:****** .*********.** *:******:: * *:*:*

LA1-ori AYRHALTVQDADEWIEGDRTDDQFRPPSSKVMLSALRKYVNHNRLYNQFYTAAQLLAQIM 300
LAlus4  AYRHAITVQDADEWIEGDRTDDQFRPPSSKVMMSALRKYVNHNRLYNQFYTAAQLLAQIM 300
LAbarr1 AYRHTAKVTEADEWLMNERPDAAFPVLSSKAMLSAIRKYVNHNRVYNHFETAAQILAQLM 298
        ****: .* :****: .:*.*  *   ***.*:**:********:**:* ****:***:*

LA1-ori MKPVPNCAEGYAWLMHDALVNIPKFGSIRGRYPFLLSGDAALIQATALEDWSAIMAKPEL 360
LAlus4  MKPVPNCAEGYAWLMHDALVNLPKFGSVRGRYPFLLAGDAALIQATALEDWSAIMAKPEL 360
LAbarr1 VKPLPNNAEGHAWLLHDPVVNIPKFGSVRGRYPFLLTGEASLVQATALEDWSAIMGKPEI 358
        :**:** ***:***:**.:**:*****:********:*:*:*:*************.***:

LA1-ori VFTYAMQVSVALNTGLYLRRVKKTGFGTTIDDSYEDGAFLQPETFVQAALACCTGQDAPL 420
LAlus4  IFTYAMQVAVALNTGLYLRRVKKTGFGTTVDDSYEDGAFLQPETFVQAAIACCTGQDAPL 420
LAbarr1 VFTYAVDHAIAVNTGLYLRRIKKTGMGTRVDDSYEDGVFLSPETFMAAAVACATGEDAPL 418
        :****:: ::*:*******:****:** :*******.**.****: **:**:**:****

LA1-ori NGMSDVYVTYPDLLEFDAVTQVPITVIEPAGYNIVDDHLVVVGVPVACSPYMIFPVAAFD 480
LAlus4  NGMSDVYVTYPDLLEFDTITRVPVTVLEPEGYNIQDGALEVTGVPIACSPYMIFPVAAFD 480
LAbarr1 NGMSDVYVFYPQFVDLDTVTYIPAQVREAEGYNVTSEGLKVLGVPLACSPVLLYPMAAFD 478
        ******** **:::::*::* :*  * *. ***: .  * * ***:**** :::*:****

LA1-ori TANPYCGNFVIKAANKYLRKGAVYDKLEAWKLAWALRVAGYDTHFKVYGDTHGLTKFYAD 540
LAlus4  EANPYSGSFVIKPALKYLRKGALYAKLEAWKLAWAMRIAGYDTEFKACGNTHGLSKFYAD 540
LAbarr1 DANPYSGSFKIGKAERYVRGRAVYSPFEAWKLAWAARIAGYDTRVSIPGDNTGLSKLYAD 538
         ****.*.* *  * :*:*  *:*  :******** *:*****...  *:. **:*:***

LA1-ori NGDTWTHIPEFVTDGDVMEVFVTAIERRARHFVELPRLNSPAFFRSVEVSTTIYDTHVQA 600
LAlus4  NSDSWTHIPEFVSDEEVTEVYVTNIERRARHFVELPRLNSPAFFKPVEVSTAIYDTYVQA 600
LAbarr1 NADSWTHIPDSFLNPEAEFIYVTDLERRRRHFIDLPNMTNPSFARSIEVQITLNDMYVES 598
        *.*:*****: . : :.  ::** :*** ***::**.:..*:* :.:**. :: * :*::
                                                      Gag◄► Pol
LA1-ori GAHAVYHASRINLDYVKPVSTGIQVINAGELKNYWGSVRRTQQGFRSGRSYDASCNAYRR 660
LAlus4  GTFSVYHASRINLDYVKPVSAGIQVINAGELRNYWGSVRRTQQGFRSGRSYDASRNAYRR 660
LAbarr1 TPGSRSRAMVLSRDYVAPIASGIQVISAADLRNYWGSVRRSNAGFILGRFYDASSNSYRK 658
         . :  :*  :. *** *:::*****.*..:*:********:: **  ** **** *:**:

LA1-ori TYSWRCPRRVDRTGGQCFSRVNVIEPSHGPRPTRYILQEPGTYPAWIRFRNRVQAVSRQK 720
LAlus4  TYSWHCPRRLDRTDGKCFSRVNIIEPSHGPRPMRYILNEPNTYPAWIRFRNRVQAVSRQR 720
LAbarr1 EYRWSRPPRGDRTRARAFSRINLIESEHGHRPRRYIASGNEVVPIWIRFGRRIECVSRQK 718
         * *  * * ***  .:.***:*:**..** ** ***  .    * **** .*::.****:
                     ► ————————— Variable region of 44 amino acids ————————— ◄
LA1-ori ATHFLFDIVPAAVISDFTTSDTSSFAYKSHTYAVNVTALRFSDTYALYVQTDTNMTILSP 780
LAlus4  ATHFLFDIVPASKIADYTTSQTATFAYRSHTYACTVTALRFGDDYGLYVQVEANMTLLSP 780
LAbarr1 ATYFLADVIPREEAINYTSANTAQVAIRRSTYTASGILVKLGDNYGFYLHVDEDMTVKTP 778
        **:** *::*       ::*:::*: .* :  **: .    :::.* *.:*::.: :**: :*

LA1-ori AARRQASATYSQVAGFCYNTPTVMDSLANILDVDRNIRPKHFKGLRLYTRSKVTAQHHTH 840
LAlus4  AARRQASATYSQVEGFCFNTPTVMDTLANILDVDRNIRPKHFKGLRTYERSKVTAQHHTH 840
LAbarr1 ALRRYASMAYSNVRGSCYNTPTIMDTLCNIFDYDPDSRPSSFKGLRSYTKSKVTGMHHTH 838
        * ** ** :**:* * *:****:**:*.**:* * : **. ***** * :****. ****

LA1-ori LRPDELVEAAAKVSPRRKYYLMCVVELLANLQVDLEAAVATILAYVLTLSEKFVPIFLDS 900
LAlus4  LRPDEVLEAAARVSPRRKYYLLCVVELLAACEVNIEAAVATIMTYVLTLDEKFIPLFLDS 900
LAbarr1 LRPQDVLSAAKKTGNSHYYYVQVAVERLASAGVTTEATVSTIMLYVLTSNDTTRALFLDS 898
```

```
         ***::::.** :..  : **:  .** **   *  **:*:**: **** .:.  .:****

LA1-ori  RAIWVGEPGPDALTARLKASSGQIKSIHTADYEPLTELFELAVLMNRGVGHVSWQAEKDH 960
LAlus4   RTIWQGSKGPEELTARLKKASGQIKSVHTADYEPLTELFELAVLMNRGVGHVSWKTEREH 960
LAbarr1  KAIWSKTDGEDELSDRLKRVSGQIKSIHTSELEPLTELFELQVLMNRGIGHVDWNGERHN 958
         ::**    * : *: ***  ******:**:: ********* ******:***.*: *:.:

LA1-ori  RLNPDVAVVDQARLYSCVRDMFEGSKQTYKYPFMTWDDYTANRWEWVPGGSVHSQYEEDN 1020
LAlus4   RENPDVANVNQQALYACVRDMFEGAKQTYDYPYMTWDDYTSSRWEWVPGGSVHSQYSEDD 1020
LAbarr1  RTNPNTVNVDQKMLYSVVRDMFEDSKQSYKYPYMTWNDYADSRWEWIPGGSVHSQYPQDD 1018
         * **:.. *:*  **: ******.:**:*.**:***:**: .****:********* :*:

LA1-ori  DYIYPGQYTRNKFITVNKMPKHKISRMIASPPEVRAWTSTKYEWGKQRAIYGTDLRSTLI 1080
LAlus4   EYIFPGQYTRNKFITVNKMPKHKIARMIASTPEVRAWTSTKYEWGKQRAIYGTDLRSTLI 1080
LAbarr1  EYIVPGLYTRNKFITVNLMPKNKLASIGASKPEVRAWTSTKYEWGKQRAIYGTDLRSTLI 1078
         :** ** ********** ***:*:: : ** *****************************

                                                        ►—A—◄
LA1-ori  TNFAMFRCEDVLTHKFPVGDQAEAAKVHKRVNMMLDGASSFCFDYDDFNSQHSIASMYTV 1140
LAlus4   TNFAMFRCEDVLTHKFPVGDQAEAAKVHKRVNMMLDGASSFCFDYDDFNSQHSISSMYTV 1140
LAbarr1  TNFAMFRCEDVLKHKFPVGDQAEASKVHKRISMMLGNASSFCFDYDDFNSQHSISSMYTV 1138
         ************.***********:*****:.***..*****************:*****

                                                             ►———B——◄
LA1-ori  LCAFRDTFSRNMSDEQAEAMNWVCESVRHMWVLDPDTKEWYRLQGTLLSGWRLTTFMNTV 1200
LAlus4   LCAFRDAFTRNMSIEQREAMDWVCESVKHMWVLDPDTKTWYQLKGTLLSGWRLTTFMNTV 1200
LAbarr1  LLAFRDAYSRNMSPAQLRAMDWVCESTKHMYAKDPGTNDWYELKGTLLSGWRLTTFMNTV 1198
         * ****:::****  *  .**:*****.:**:. **.*: **.*:****************

                                   ►C◄
LA1-ori  LNWAYMKLAGVFDLDDVQDSVHNGDDVMISLNRVSTAVRIMDAMHRINARAQPAKCNLFS 1260
LAlus4   LNWAYKKLAGVFDLDDVQDSVHNGDDVMISLNRVSTAVRIMDAMHRINARAQPAKCNLFS 1260
LAbarr1  LNWAYMKVAGVFDITDVEDSVHNGDDVMISLNRVSTAVKIMERMRLINARAQEAKCNLFS 1258
         ***** *:*****: **:*********************:**: *: ****** *******

           ►D◄
LA1-ori  ISEFLRVEHGMSGGDGLGAQYLSRSCATLVHSRIESNEPLSVVRVMEADQARLRDLANRT 1320
LAlus4   ISEFLRVEHGMSGGDGLGAQYLSRSCATLVHSRIESNEPLSVVRVMEADKTRLRDLANRT 1320
LAbarr1  ISEFLRVEHGMTGKSGLGAQYLSRSCATIVHSRIESNEPISLVRLLEADKTRLRDLSDRT 1318
         ***********:* .*************:**********:*:**::***::*****::**

LA1-ori  RVQSAVTAIKEQLDKRVTKIFGVGDDVVRDIHTAHRVCGGISTDTWAPVETKIITDNEAY 1380
LAlus4   NIKASVTEIEEQLDRRVTSIFKVDREVVKAISTAHRVCGGISTDPWAPVTTKIKTDNEAY 1380
LAbarr1  TREDVLVNIKYELDMRAVNVFGADYNVVQDIYKSHRVCGGINDDKWGSVDTEIHTDSGAY 1378
           :  :. *: :** *...:* .. :**: *  .:*******. * *..* *:* **. **

LA1-ori  EIPYEIDDPSFWPGVNDYAYKVWKNFGERLEFNKIKDAVARGSRSTIALKRKARITSKKN 1440
LAlus4   EIPYEIDDPSFWPGVNDYAYKVWQNFGERLEFNKIKDAVSKGSRNTIALKRKAKISAVKN 1440
LAbarr1  QIPEEIDDPSFWPGVNDYARKAYNILGEKLEFNKIKSAVARGSRLTIAMKRRARVGSTPT 1438
         :** *************** *.:: :**:*******.**::*** ***:**:*:: :  .

LA1-ori  EFANKSEWERTMYKAYKGLAVSYYANLSKFMSIPPMANIEFGQARYAMQAALDSSDPLRA 1500
LAlus4   DFVNKSEWERTMYKAYKGLAVSYYANLSKFMSIPPMANIEFGQARFAMQAALDSSDPLRA 1500
LAbarr1  EFINIKQWERAMYRAFKGVAISYYTSLSKFMAVPPISGLDRGEGAMAVNAAIASADPLRA 1498
         :* *  .:***:**:*:**:*:***:.*****::**::.:: *:.  *::**: *:*****

LA1-ori  LQVIL 1505
LAlus4   LQIFL 1505
LAbarr1  LQILV 1503
         **:::
```

Figure S2. Comparison of the amino acid sequences of Gag-Pol encoded by ScV-LA1-original, ScV-LAlus4, and TdV-LAbarr1 dsRNA genomes. The separation between Gag and Pol domains is indicated (Gag ◄► Pol). The H154 residue required for 5′cap-snatching is black shaded. The stretch of variable amino acid sequence located downstream from H154 is double underlined. The four crucial residues for cap recognition (Tyr-150, Asp-152, Tyr-452, and Tyr-538) are grey shaded. A variable region of 44 amino acids in the N-terminal region of Pol that is likely to separate Gag and Pol is indicated above the sequence. The highly conserved RdRp domain located in the central third of Pol is underlined, the four consensus motifs (A-D) conserved in RNA-dependent RNA polymerases from totiviruses are indicated above the sequence, and the conserved amino acids for each motif are grey shaded. Methionine (M) from in-frame re-initiation codons

(AUG) in the N-terminal region of Pol are shown in bold. Asterisks (*) indicate identical amino acids; colons (:) and single dots (.) indicate conserved and semi-conserved amino acids, respectively.

TdV-LAbarr1	100	61	62	62	62	62	62	62	62	62	62	61	63	62	62	62	62	62	62	63
SkV-LA1082	61	100	73	74	73	73	73	74	74	74	74	74	74	74	73	74	73	73	74	74
ScV-LA1-original	62	73	100	74	74	74	74	74	74	77	75	74	74	74	74	74	75	74	75	74
ScV-LA2-8F13	62	74	74	100	78	78	78	78	78	75	76	76	76	76	76	76	76	76	75	76
ScV-LA2-EX1125	62	73	74	78	100	98	98	98	98	75	76	75	76	76	76	76	76	76	76	76
ScV-LA1-EX231	62	73	74	78	98	100	100	98	99	75	77	75	76	76	76	76	76	76	76	76
ScV-LAlus-EX1160	62	73	74	78	98	100	100	98	99	75	77	75	76	76	76	76	76	76	76	76
ScV-LAlus1-EX436	62	74	74	78	98	98	98	100	99	75	77	75	76	76	76	76	76	77	76	76
ScV-LAlus4-EX229	62	74	74	78	98	99	99	99	100	75	76	75	76	76	76	76	76	76	76	76
SpV-LA45	62	74	76	75	75	75	75	75	75	100	77	77	77	76	76	76	77	76	77	77
SkV-LAFM1183	62	74	75	76	76	77	77	77	76	77	100	79	79	80	79	79	79	79	79	79
SpV-LA4650	61	74	74	76	75	75	75	75	75	77	79	100	90	90	90	90	90	82	82	83
SpV-LA62	63	74	74	76	76	76	76	76	76	77	79	90	100	92	90	91	91	82	82	83
SpV-LA1143	62	74	74	76	76	76	76	76	76	76	80	90	92	100	90	92	92	82	82	82
SuV-LA10560	62	73	74	76	76	76	76	76	76	76	79	90	90	90	100	92	92	82	82	82
SpV-LA74	62	74	74	76	76	76	76	76	76	76	79	90	91	92	92	100	93	82	82	82
SpV-LA1939	62	73	75	76	76	76	76	76	76	77	79	90	91	92	92	93	100	82	82	82
SpV-LA28	62	73	74	76	76	76	76	77	76	76	79	82	82	82	82	82	82	100	90	90
SpV-LA66	62	74	75	75	76	76	76	76	76	77	79	82	82	82	82	82	82	90	100	90
SpV-LA21	63	74	74	76	76	76	76	76	76	77	79	83	83	82	82	82	82	90	90	100

Colours: *S. cerevisiae* cluster; Close to *S. cerevisiae* cluster; *S. paradoxus* cluster; Close to *S. paradoxus* cluster; Equally distant from both clusters (74-75% identity); Equally distant from both clusters (73-74% identity); Equally distant from both clusters (61-63% identity)

Figure S3. Percentage identity matrix for canonical V-LA nucleotide sequences. Each value was rounded to the nearest whole number.

TdV-LAbarr1	100	83	82	84	85	85	85	85	84	83	83	84	84	84	83	84	84	84	84	85
SkV-LA1082	83	100	93	93	93	93	93	93	93	93	93	92	93	93	93	93	93	93	92	92
ScV-LA1-original	82	93	100	95	95	95	95	95	95	94	94	94	94	94	93	94	94	94	94	94
ScV-LA2-8F13	84	93	95	100	96	96	96	96	96	94	95	94	95	95	94	95	95	95	94	95
ScV-LA1-EX231	85	93	95	96	100	100	100	100	100	95	94	94	95	95	94	95	95	95	96	96
ScV-LAlus-EX1160	85	93	95	96	100	100	100	100	100	95	94	94	95	95	94	95	95	95	96	96
ScV-LA2-EX1125	85	93	95	96	100	100	100	100	100	95	94	94	95	95	94	95	95	95	96	96
ScV-LAlus1-EX436	85	93	95	96	100	100	100	100	100	95	94	94	95	95	94	95	95	95	96	96
LAlus4-EX229	84	93	95	96	100	100	100	100	100	94	94	94	94	94	94	94	94	95	95	95
SpV-LA45	83	93	94	94	95	95	95	95	95	100	96	96	99	95	96	95	95	96	96	96
SkV-LAFM1183	83	93	94	95	94	94	94	94	94	96	100	97	97	97	99	97	97	97	96	97
SpV-LA28	84	92	94	94	94	94	94	94	94	96	97	100	99	99	99	98	98	98	98	98
SpV-LA66	84	93	94	95	95	95	95	95	94	96	97	99	100	99	98	98	98	99	99	98
SpV-LA21	84	93	94	95	94	95	95	95	94	95	97	99	99	100	99	99	99	99	99	99
SpV-LA1939	83	93	93	94	94	94	94	94	94	96	99	99	98	99	100	99	99	99	98	98
SpV-LA62	84	93	94	95	95	95	95	95	94	95	97	98	98	99	99	100	100	100	99	99
SpV-LA4650	84	93	94	95	95	95	95	95	94	95	97	98	98	99	99	100	100	100	99	99
SpV-LA74	84	93	94	95	95	95	95	95	95	96	97	98	99	99	99	100	100	100	99	99
SpV-LA1143	84	92	94	94	96	96	96	96	95	96	96	98	99	99	98	99	99	99	100	99
SuV-LA10560	85	92	94	95	96	96	96	96	95	96	97	98	98	99	98	99	99	99	99	100

Colours: *S. cerevisiae* cluster; Close to *S. cerevisiae* cluster; *S. paradoxus* cluster; Close to *S. paradoxus* cluster; Equally distant from both clusters (93-95% identity); Equally distant from both clusters (92-93% identity); Equally distant from both clusters (82-85% identity)

Figure S4. Percentage identity matrix for highly-conserved RdRp-domain amino-acid sequences. Each value was rounded to the nearest whole number except for those between ScV-LA1-EX231, ScV-LAlusA-EX1160, ScV-LA2-EX1125, and ScV-LAlus1-EX436 of the *S. cerevisiae*-cluster, and SpV-LA62-vs-SpV-LA4650 of the *S. paradoxus*-cluster, which initially were 100%.

TdV-LAbarr1	100	47	45	47	45	45	44	44	46	45	44	46	44	43	44	46	46	46	46	46
SkV-LA1082	47	100	68	75	75	76	75	73	74	75	72	75	76	74	74	79	79	79	79	78
SpV-LA45	45	68	100	85	82	78	77	79	78	79	78	81	79	74	77	76	76	75	75	76
SkV-LAFM1183	47	75	85	100	87	86	85	85	83	85	82	87	84	76	76	79	79	80	80	79
SpV-LA62	45	75	82	87	100	94	92	94	95	95	89	92	92	75	78	79	79	79	79	78
SuV-LA10560	44	76	78	86	94	100	93	95	93	95	86	90	90	76	76	78	78	78	78	77
SpV-LA74	44	75	77	85	92	93	100	95	94	95	86	90	90	78	75	78	78	78	78	77
SpV-LA4650	44	73	79	85	94	95	95	100	95	98	88	90	92	76	76	78	78	79	79	78
SpV-LA1143	46	74	78	83	95	93	94	95	100	98	87	89	91	75	78	78	78	78	78	78
SpV-LA1939	45	75	79	85	95	95	95	98	98	100	87	89	91	76	78	79	79	79	79	78
SpV-LA66	44	72	78	82	89	86	86	89	87	87	100	92	93	74	73	76	76	76	76	75
SpV-LA28	46	75	81	87	92	90	90	90	89	89	92	100	95	78	77	79	79	79	79	78
SpV-LA21	44	76	79	84	92	90	90	92	91	91	93	95	100	78	78	82	82	82	86	81
ScV-LA1-original	43	74	74	76	75	76	75	76	75	76	74	78	78	100	76	80	80	80	80	81
ScV-LA2-8F13	44	74	77	76	78	76	75	76	78	78	73	77	78	76	100	85	85	83	83	84
ScV-LAlus4-EX229	46	79	76	79	79	78	78	78	78	79	76	79	82	80	85	100	100	98	98	99
ScV-LAlus1-EX436	46	79	76	79	79	78	78	78	78	79	76	79	82	80	85	100	100	98	98	99
ScV-LA1-EX231	46	79	75	80	79	78	78	79	78	79	76	79	82	80	83	98	98	100	100	99
ScV-LAlus-EX1160	46	79	75	80	79	78	78	79	78	79	76	79	82	80	83	98	98	100	100	99
ScV-LA2-EX1125	46	78	76	79	78	77	77	78	78	78	75	78	81	81	84	99	99	99	99	100

Colours: *S. cerevisiae* cluster; Close to *S. cerevisiae* cluster
S. paradoxus cluster; Close to *S. paradoxus* cluster
Equally distant from both clusters (74-85% identity)
Equally distant from both clusters (72-79% identity)
Equally distant from both clusters (43-47% identity)

Figure S5. Percentage identity matrix for Gag amino-acid sequences of LA viruses. Each value was rounded to the nearest whole number.

TdV-LAbarr1	100	20	34	32	32	32	32	32	30	25	30	27	23	25	25	25	23	27	25	27
ScV-LA1-original	20	100	57	64	64	64	64	64	68	61	64	64	64	66	66	64	68	64	57	55
SkV-LA1082	34	57	100	68	68	68	68	68	59	57	55	57	57	57	57	57	59	57	50	57
ScV-LA1-EX231	32	64	68	100	100	100	100	100	84	68	68	70	66	70	70	66	70	68	64	68
ScV-LAlus-EX1160	32	64	68	100	100	100	100	100	84	68	68	70	66	70	70	66	70	68	64	68
ScV-LA2-EX1125	32	64	68	100	100	100	100	100	84	68	68	70	66	70	70	66	70	68	64	68
ScV-LAlus4-EX229	32	64	68	100	100	100	100	100	84	68	68	70	66	70	70	66	70	68	64	68
ScV-LAlus1-EX436	32	64	68	100	100	100	100	100	84	68	68	70	66	70	70	66	70	68	64	68
ScV-LA2-8F13	30	68	59	84	84	84	84	84	100	68	73	70	68	73	73	66	70	68	68	66
SpV-LA74	25	61	57	68	68	68	68	68	68	100	91	91	91	95	95	80	86	82	68	77
SpV-LA1143	30	64	55	68	68	68	68	68	73	91	100	95	91	95	95	82	89	84	70	75
SpV-LA62	27	64	57	70	70	70	70	70	70	91	95	100	91	95	95	86	89	86	73	80
SuV-LA10560	23	64	57	66	66	66	66	66	68	91	91	91	100	95	95	80	86	82	68	77
SpV-LA4650	25	66	57	70	70	70	70	70	73	95	95	95	95	100	100	84	91	86	73	80
SpV-LA1939	25	66	57	70	70	70	70	70	73	95	95	95	95	100	100	84	91	86	73	80
SpV-LA66	25	64	57	66	66	66	66	66	66	80	82	86	80	84	84	100	93	91	70	77
SpV-LA21	23	68	59	70	70	70	70	70	70	86	88	89	86	91	91	93	100	91	68	75
SpV-LA28	27	64	57	68	68	68	68	68	68	82	84	86	82	86	86	91	91	100	73	80
SpV-LA45	25	57	50	64	64	64	64	64	68	68	70	73	68	73	73	70	68	73	100	80
SkV-LAFM1183	27	55	57	68	68	68	68	68	66	77	75	80	77	80	80	77	75	80	80	100

Colours: *S. cerevisiae* cluster; Close to *S. cerevisiae* cluster
S. paradoxus cluster; Close to *S. paradoxus* cluster
Equally distant from both clusters (64-68% identity)
Equally distant from both clusters (50-68% identity)
Equally distant from both clusters (23-32% identity)

Figure S6. Percentage identity matrix for the variable hydrophobic 44-amino-acid stretch of Pol sequences. Each value was rounded to the nearest whole number, except for the *S. cerevisiae*-cluster and SpV-LA4650-vs-SpV-LA1939 values that initially were 100%.

TdV-LAbarr1	100	17	17	11	22	17	17	17	17	17	28	22	28	33	22	22	22	22	22	11
SkV-LA1082	17	100	68	63	63	47	53	53	58	58	53	53	53	58	53	53	53	53	53	58
ScV-LA1-original	17	68	100	58	47	42	53	53	47	47	42	42	42	53	42	42	42	42	42	47
SpV-LA45	11	63	58	100	47	42	47	47	42	47	37	37	37	47	63	63	63	63	63	58
SpV-LA62	22	63	47	47	100	79	79	79	89	84	79	74	68	63	68	68	68	68	68	68
SpV-LA4650	17	47	42	42	79	100	84	84	89	84	63	74	68	58	58	58	58	58	58	58
SuV-LA10560	17	53	53	47	79	84	100	100	89	95	68	79	74	58	63	63	63	63	63	63
SpV-LA1939	17	53	53	47	79	84	100	100	89	95	68	79	74	58	63	63	63	63	63	63
SpV-LA1143	17	58	47	42	89	89	89	89	100	95	74	84	79	58	58	58	58	58	58	63
SpV-LA74	17	58	47	47	84	84	95	95	95	100	74	84	79	58	63	63	63	63	63	68
SpV-LA66	28	53	42	37	79	63	68	68	74	74	100	74	84	58	58	58	58	58	58	58
SpV-LA28	22	53	42	37	74	74	79	79	84	84	74	100	89	58	47	47	47	47	47	53
SpV-LA21	28	53	42	37	68	68	74	74	79	79	84	89	100	58	47	47	47	47	47	53
ScV-LA2-8F13	33	58	53	47	63	58	58	58	58	58	58	58	58	100	74	74	74	74	74	63
ScV-LA1-EX231	22	53	42	63	68	58	63	64	58	63	58	47	47	74	100	100	100	100	100	74
ScV-LAlus-EX1160	22	53	42	63	68	58	63	63	58	63	58	47	47	74	100	100	100	100	100	74
ScV-LA2-EX1125	22	53	42	63	68	58	63	63	58	63	58	47	47	74	100	100	100	100	100	74
ScV-LAlus4-EX229	22	53	42	63	68	58	63	63	58	63	58	47	47	74	100	100	100	100	100	74
ScV-LAlus1-EX436	22	53	42	63	68	58	63	63	58	63	58	47	47	74	100	100	100	100	100	74
SkV-LAFM1183	11	58	47	58	68	58	63	63	63	68	58	53	53	63	74	74	74	74	74	100

Colours: *S. cerevisiae* cluster; Close to *S. cerevisiae* cluster
S. paradoxus cluster; Close to *S. paradoxus* cluster
Equally distant from both clusters (53-58% identity)
Equally distant from both clusters (42-53% identity)
Equally distant from both clusters (17-22% identity)

Figure S7. Percentage identity matrix for the variable 19-amino-acid stretch located downstream of H153/154 of Gag sequences. Each value was rounded to the nearest whole number, except for the *S. cerevisiae*-cluster that initially was 100%.
